# Structural visualization of transcription activated by a multidrug-sensing MerR family regulator

**DOI:** 10.1038/s41467-021-22990-8

**Published:** 2021-05-11

**Authors:** Yang Yang, Chang Liu, Wei Zhou, Wei Shi, Ming Chen, Baoyue Zhang, David G. Schatz, Yangbo Hu, Bin Liu

**Affiliations:** 1 Roy J. Carver Department of Biochemistry, Biophysics and Molecular Biology, Iowa State University, Ames, IA USA; 2 Department of Molecular, Cellular and Developmental Biology, Yale University, New Haven, CT USA; 3 Howard Hughes Medical Institute, Yale University, New Haven, CT USA; 4 Department of Immunobiology, Yale School of Medicine, New Haven, CT USA; 5 Key Laboratory of Special Pathogens and Biosafety, Wuhan Institute of Virology, Center for Biosafety Mega-Science, Chinese Academy of Sciences, Wuhan, China; 6 University of Chinese Academy of Sciences, Beijing, China; 7 Section of Transcription & Gene Regulation, The Hormel Institute, University of Minnesota, Austin, MN USA

**Keywords:** Enzyme mechanisms, Bacterial transcription, Transcription, Cryoelectron microscopy, X-ray crystallography

## Abstract

Bacterial RNA polymerase (RNAP) holoenzyme initiates transcription by recognizing the conserved –35 and –10 promoter elements that are optimally separated by a 17-bp spacer. The MerR family of transcriptional regulators activate suboptimal 19–20 bp spacer promoters in response to myriad cellular signals, ranging from heavy metals to drug-like compounds. The regulation of transcription by MerR family regulators is not fully understood. Here we report one crystal structure of a multidrug-sensing MerR family regulator EcmrR and nine cryo-electron microscopy structures that capture the EcmrR-dependent transcription process from promoter opening to initial transcription to RNA elongation. These structures reveal that EcmrR is a dual ligand-binding factor that reshapes the suboptimal 19-bp spacer DNA to enable optimal promoter recognition, sustains promoter remodeling to stabilize initial transcribing complexes, and finally dissociates from the promoter to reverse DNA remodeling and facilitate the transition to elongation. Our findings yield a comprehensive model for transcription regulation by MerR family factors and provide insights into the transition from transcription initiation to elongation.

## Abstract (web summary)

The MerR family of transcriptional regulators, such as EcmrR, activate promoters with a structure that is suboptimal for recognition by RNA polymerase holoenzyme. Structural insights into the EcmrR-dependent transcription process elucidate the mechanisms enabling optimal promoter recognition and transition from initiation to elongation.

## Introduction

Bacterial transcription is initiated from the recognition of core promoter sequence by a σ factor in the RNAP holoenzyme (α_2_ββ'ωσ), mediated by specific binding of σ_4_ and σ_2_ domains to the conserved –35 and –10 promoter elements^1–3^. For promoters recognized by the primary σ factor, σ^70^ or σ^A^, the spacer region between the –35 and –10 elements is variable in sequence but has a consensus length of 17 ± 1 bp^4^. Transcription from promoters with spacers that significantly deviate from the canonical 17-bp length is exceedingly inefficient without the aid of additional regulators^5^.

MerR family transcriptional regulators bind to the primary σ factor-dependent promoters containing 19- or 20-bp spacers and activate transcription of downstream genes, many of which encode efflux pumps^6–9^. The extra 2–3 bp in the spacers not only place the −35 and −10 elements farther apart, but also alter their relative orientation along the DNA axis^10–12^, preventing optimal promoter recognition and subsequent open complex formation. MerR family regulators have conserved N-terminal DNA-binding domains (NTDs) that recognize pseudo-palindromic sequences between the –35 and –10 elements^10–13^. Their C-terminal effector-binding domains (CTDs) are highly divergent and sense a variety of cellular signals. The NTDs and CTDs are connected by a long central helix that dimerizes into an antiparallel coiled coil. Binding of effectors induces allosteric conformational changes in the NTDs, resulting in promoter remodeling^10–13^.

Structures of BmrR and CueR, two MerR family transcriptional regulators that respond to cationic lipophilic drug-like compounds^14^ and Cu(I)^15^, respectively, revealed that these regulators under-twist and sharply bend the spacer DNA when they are activated by binding to their effectors^11,12^. This local modulation of DNA structure results in shortened distance and rotated angles between the –35 and –10 elements that become readily recognizable by the σ factor. Such DNA distortion mechanism was further confirmed by the recent cryo-EM structures of CueR and BmrR transcription activation complexes (CueR-TAC and BmrR-TAC, respectively)^16,17^, which were reported during the review process of our study. However, the steps following open conformation formation on MerR family regulators-dependent promoters remain largely uncharacterized.

Here we describe a series of crystal and cryo-EM structures of a multidrug-sensing MerR family transcriptional regulator in *Escherichia coli* (hereinafter referred to as EcmrR) and its complexes with *E. coli* RNAP at multiple steps during transcription including open complex formation, initial transcription, promoter escape, and elongation complex formation; at resolutions of 1.4–3.3 Å. (Supplementary Fig. 1a–e, Supplementary Figs. 2–5; Table 1 and Supplementary Table 1). These structures, in combination with supporting biochemical and in vivo transcription analyses, reveal two possible multidrug-binding sites in EcmrR and the molecular details of promoter remodeling by EcmrR with ligand binding in one or both of its multidrug-binding sites. In addition, we observed a previously uncharacterized interaction between EcmrR and σ^70^ that helps stabilize EcmrR-promoter binding during transcription initiation but is broken prior to promoter escape, although the extent to which this interaction influences transcription activation by EcmrR remains to be determined. Our study elucidates the dynamic process of EcmrR-regulated transcription in detail and suggests a common mechanism utilized by MerR family factors for transcription regulation.

**Table 1:** Cryo-EM data collection, refinement and validation statistics.

	EcmrR-RP_o_ (EMDB-22234) (PDB 6XL5)	EcmrR-RP_o_ (clear NCR-EcmrR interface density) (EMDB-23291)	EcmrR/DNA (RP_o_) (EMDB-22235) (PDB 6XL6)	EcmrR-RP_itc-3nt_ (EMDB-22236) (PDB 6XL9)	EcmrR/DNA (RP_itc-3nt_) (EMDB-22237) (PDB 6XLA)	EcmrR-RP_itc-4nt_ (EMDB-22245) (PDB 6XLJ)	EcmrR/DNA (RP_itc-4nt_) (EMDB-22246) (PDB 6XLK)	RP_itc-5nt_ (EMDB-22247) (PDB 6XLL)	RD_e1_ (EMDB-22248) (PDB 6XLM)	RD_e2_ (EMDB-22249) (PDB 6XLN)
**Data collection and processing**										
Magnification	105,000	105,000	105,000	96,000	96,000	105,000	105,000	105,000	105,000	105,000
Voltage (kV)	300	300	300	300	300	300	300	300	300	300
Electron exposure (e–/Å^2^)	50.68	50.68	50.68	30.00	30.00	50.46	50.46	50.46	50.46	50.46
Defocus range (μm)	1.2–2.2	1.2–2.2	1.2–2.2	0.8–2.6	0.8–2.6	1.2–2.2	1.2–2.2	1.2–2.2	1.2–2.2	1.2–2.2
Pixel size (Å)	0.8887	1.333	1.333	0.895	0.895	1.333	1.333	1.333	1.333	1.333
Symmetry imposed	C1	C1	C1	C1	C1	C1	C1	C1	C1	C1
Initial particle images (no.)	249,694	249,694	249,694	446,920	446,920	372,776	372,776	372,776	372,776	372,776
Final particle images (no.)	214,970	48,299	214,970	110,796	110,796	80,448	80,448	126,197	21,651	76,916
Map resolution (Å)	2.5	2.9	3.0	2.5	3.1	2.7	3.3	2.7	3.2	2.8
FSC threshold	0.143	0.143	0.143	0.143	0.143	0.143	0.143	0.143	0.143	0.143
Map resolution range (Å)	2.0–4.5	2.5–5.2	2.7–4.2	2.0–4.5	2.5–4.5	2.7–4.7	2.7–4.7	2.7–4.7	2.7–5.7	2.7–4.7
**Refinement**										
Initial model used (PDB code)	6OUL		6WL5	6OUL	6WL5	6OUL	6WL5	6OUL	6OUL	6OUL
Model resolution (Å)	2.7		3.2	2.8	3.2	3.0	3.5	2.9	3.5	3.0
FSC threshold	0.5		0.5	0.5	0.5	0.5	0.5	0.5	0.5	0.5
Model resolution range (Å)	53.1–2.3		29.5–2.9	59.8–2.5	25.0–2.8	56.0–2.6	29.2–3.2	43.9–2.6	51.6–3.0	39.4–2.7
Map sharpening *B* factor (Å^2^)	−69.4		−50.0	−74.2	−70.0	−61.8	−50.0	−68.5	−43.0	−63.0
Model composition										
Non-hydrogen atoms	35830		5456	35113	5403	35904	5456	31136	28246	25958
Protein residues	4220		536	4137	536	4221	536	3683	3485	3207
Nucleotides	108		46	110	46	111	46	102	40	40
Ligands	10		4	8	2	13	4	6	5	5
*B* factors (Å^2^)										
Protein	76.67		65.38	114.42	59.18	108.95	74.53	104.60	123.64	102.22
Nucleotide	83.29		62.01	127.31	44.17	115.15	82.18	160.80	125.88	109.97
Ligand	119.25		79.32	143.27	81.38	139.45	91.86	94.54	108.04	95.00
R.m.s. deviations										
Bond lengths (Å)	0.011		0.005	0.008	0.005	0.014	0.008	0.009	0.008	0.012
Bond angles (°)	1.195		0.890	1.181	0.901	1.283	0.970	1.128	1.060	1.180
Validation										
MolProbity score	1.41		1.28	1.32	1.13	1.46	1.31	1.40	1.50	1.47
Clashscore	7.51		5.23	5.81	3.37	8.47	5.71	7.34	9.42	8.79
Poor rotamers (%)	0.00		0.00	0.00	0.00	0.16	0.41	0.06	0.07	0.22
Ramachandran plot										
Favored (%)	99.17		100.00	98.91	99.81	98.28	99.06	98.99	99.28	99.03
Allowed (%)	0.83		0.00	1.04	0.19	1.69	0.94	1.01	0.69	0.94
Disallowed (%)	0.00		0.00	0.05	0.00	0.02	0.00	0.00	0.03	0.03

## Results

### EcmrR activates transcription in response to multidrug binding

**Fig. 1:**
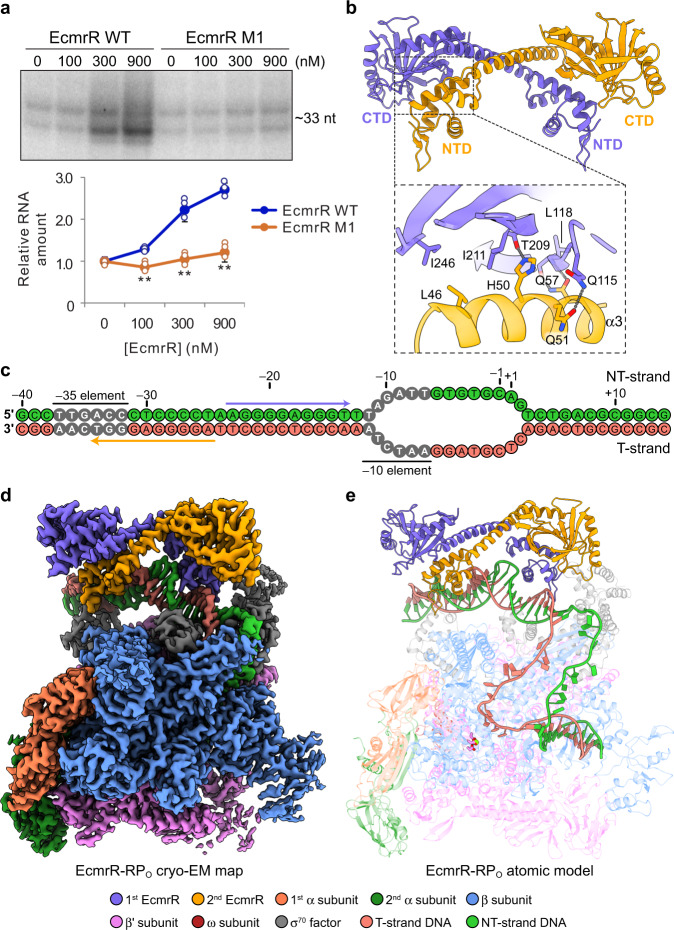
Structural and functional overview of EcmrR. **a** Activation of in vitro transcription from a synthetic template DNA with a 19-bp promoter spacer by wild-type EcmrR (EcmrR WT) or EcmrR bearing mutations in the cross-subunit NTD-CTD interface (L46A/H50A/Q57A, designated EcmrR M1). The concentrations (in nM) of EcmrR WT and EcmrR M1 are indicated. The experiment was repeated three times and similar results were obtained. RNA products were quantified from these three independent experiments and are shown as mean ± SD. Both of the two distinct RNA bands were quantified, and their signals are combined for plotting. Statistical analyses were performed using the unpaired Student’s *t*-test (two-tailed). **P < 0.01. **b** Structure of EcmrR dimer. The model was extracted from the cryo-EM structure of EcmrR-RP_o_. Cross-subunit NTD-CTD interactions are shown in the dashed box. Interacting residues are shown as sticks. Hydrogen bonds are shown as gray dotted lines. **c** Schematics of synthetic promoter DNA scaffolds in EcmrR-RP_o_. The two halves of the palindromic sequence recognized by EcmrR dimer are highlighted by medium blue and orange arrows, respectively. NT-strand, non-template strand; T-strand, template strand. **d** Cryo-EM map of EcmrR-RP_o_. The map was generated by merging the consensus map of the full EcmrR-RP_o_ complex and the focused map of EcmrR-spacer DNA subcomplex from EcmrR-RP_o_ (Supplementary Fig. 2) using the Combine Focused Maps tool in Phenix^46^. **e** Overall cryo-EM structure of EcmrR-RP_o_. Three catalytic aspartate residues (β' D460, β' D462 and β' D464) and the Mg^2+^ ion in the polymerase active center are shown as violet sticks and green sphere, respectively. Source data are provided as a Source Data file.

EcmrR shares high sequence similarity with BmrR in the N-terminal ~110 amino acids, which contain the conserved helix-turn-helix (HTH) motif for DNA binding and a characteristic long dimerization helix (α5) (Supplementary Fig. 1a). The NTD and the dimerization helix are connected by a hinge loop, which was shown to be the main allosteric link between NTD and CTD^11^ (Supplementary Fig. 1a). Although the CTDs of EcmrR (residues 112–269) and BmrR are highly divergent in sequence, their three-dimensional structures are readily superimposable (Supplementary Fig. 1a, e, f). Both CTDs belong to GyrI-like domain family, a well-characterized motif that is adapted for multidrug binding^18^. In addition to its structural similarity with BmrR, EcmrR is also able to activate the transcription from a promoter with a suboptimal 19-bp spacer between the –35 and –10 elements both in vitro and in vivo (Fig. 1a and Supplementary Fig. 1g). Moreover, the transcription activation is further boosted by a range of structurally diverse drug-like compounds, indicating broad selectivity of ligand binding by EcmrR (Supplementary Fig. 1h).

Like BmrR, full-length EcmrR forms a homodimer through the dimerization helix and cross-subunit NTD-CTD interactions (Fig. 1b), although the interaction surfaces on their CTDs differ in the two proteins (Supplementary Fig. 1i). Mutation of the residues (L46, H50 and Q57) at the EcmrR cross-subunit NTD-CTD binding interface interferes with EcmrR dimer formation and significantly decreases its transcription activation activity (Fig. 1a and Supplementary Fig. 1g). Taken together, these structural observations and functional data show that EcmrR is a multidrug-sensing MerR family regulator and activates transcription from a suboptimal promoter.

### EcmrR facilitates promoter recognition by RNAP

**Fig. 2:**
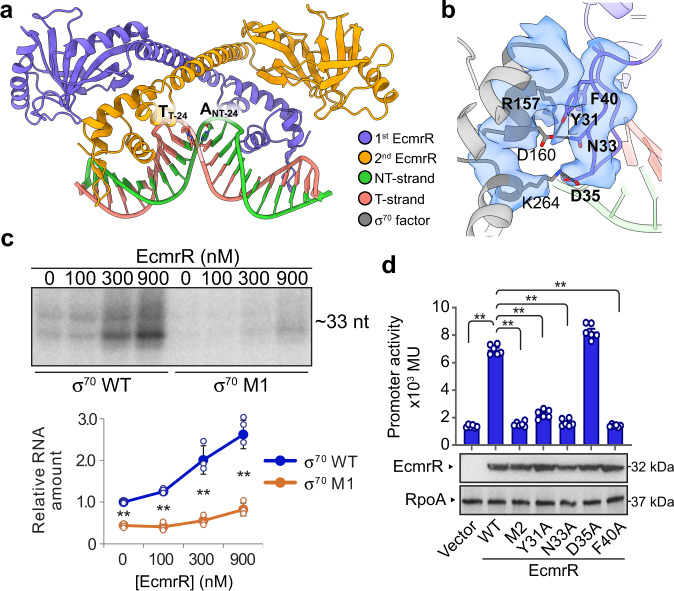
EcmrR interacts with σ^70^ NCR in EcmrR-RNAP-promoter complex. **a** Structure of EcmrR-promoter spacer subcomplex from EcmrR-RP_o_. The unpaired adenosine and thymidine at the center of the spacer are labeled and shown as sticks. **b** Detailed interactions between EcmrR NTD and σ^70^ NCR. The contacting residues at the EcmrR NTD and σ^70^ NCR interface are superimposed with the cryo-EM densities (blue surfaces) contoured at 6σ. Hydrogen bonds and salt bridges are shown as gray dotted lines. **c** In vitro transcription from an EcmrR-dependent promoter using *E.coli* RNAP assembled with wild-type σ^70^ (σ^70^ WT) or σ^70^ bearing mutations in the EcmrR NTD-interacting interface (R157A/D160A/K264A, designated σ^70^ M1). The concentrations (in nM) of EcmrR are indicated. The experiment was repeated three times and similar results were obtained. RNA products were quantified from these three independent experiments and are shown as mean ± SD. Both of the two distinct RNA bands were quantified, and their signals are combined for plotting. **d** Activation of in vivo transcription from an EcmrR-dependent promoter by EcmrR WT and EcmrR bearing mutations in the σ^70^ NCR-interacting interface (Y31A, N33A, D35A, F40A, or the combination of all four mutations, designated EcmrR M2). The promoter is fused to a β-galactosidase reporter gene (*lacZ*). Promoter activity was measured by β-galactosidase activity in miller units (MU). Expression levels of EcmrR and the loading control RpoA are shown. Data were obtained from three colonies performed in duplicate and are shown as mean ± SEM. Statistical analyses were performed using the unpaired Student’s *t*-test (two-tailed). **P < 0.01. Source data are provided as a Source Data file.

To initiate transcription, bacterial RNAP holoenzyme locates promoter DNA through specific interactions with the –35 and –10 elements and unwinds about 13 bp of the DNA duplex to form the transcription-competent RNAP-promoter open complex (RP_o_)^19,20^. To understand how EcmrR facilitates promoter recognition by RNAP and subsequent formation of RP_o_, we assembled an RNAP-EcmrR complex on a heteroduplex DNA scaffold containing a 19-bp spacer between the –35 and –10 elements (Fig. 1c and Supplementary Fig. 1b) in the presence of tetraphenylantimonium (TPSb^+^), a TPP^+^ analog that was shown to up-regulate BmrR-mediated transcription^12^. The resulting 2.5 Å cryo-EM structure of EcmrR-RP_o_ contains a 13-nt transcription bubble with template strand (T-strand) DNA positioned at the polymerase catalytic center for RNA synthesis (Fig. 1c–e and Supplementary Fig. 2). An EcmrR dimer binds to promoter DNA between the –35 and –10 elements and introduces a 58° kink in the DNA backbone, facilitated by A:T base unpairing at the center of the pseudo-palindromic sequence recognized by the EcmrR dimer (Fig. 2a, Supplementary Fig. 6 and Supplementary Video 1). As a result, the linear distance between the –35 and –10 elements is reduced to 54.4 Å, close to that in a promoter with a 17-bp spacer (52.8 Å) during transcription initiation^21^ (Supplementary Fig. 6a). In addition, EcmrR under-twists the 19-bp spacer DNA by 82° relative to an ideal B-form 19-bp DNA and realigns the –35 and –10 elements for recognition by σ_4_ and σ_2_ domains, respectively (Supplementary Fig. 6a). The spacer DNA conformation in EcmrR-RP_o_ is highly similar to those observed in the structures of BmrR-TAC and CueR-TAC (Supplementary Fig. 7), reflecting a shared DNA remodeling mechanism by MerR family regulators. Whereas spacer DNA remodeling are almost identical in the structures of stand-alone CueR-DNA complex and CueR-TAC^16^, the DNA backbone kinks and under-twists in the BmrR-DNA complex structure lacking RNAP are milder (48° and 69°) compared with those in the BmrR-TAC structure, leading to insufficient shortening of the spacer region (56.6 Å) and a suboptimal phase angle between –35 and –10 elements^12,17^ (Supplementary Fig. 6a). While the relatively milder DNA distortion in the BmrR-DNA complex structure could be affected by crystal packing, it is also possible that for promoters recognized by a subset of MerR family regulators, such as BmrR, RNAP contributes to promoter remodeling for optimal positioning of the –35 and –10 elements during open complex formation.

EcmrR-promoter binding is mediated by both base-specific hydrogen bonds and backbone interactions (Supplementary Fig. 6b). The characteristic HTH motif and a following β-hairpin straddle the DNA backbone. K16 from α2 and Y38 from the β1–β2 loop insert into the major and minor grooves, respectively, and make base-specific hydrogen bonds. Backbone phosphate interactions are mainly contributed by H19, Y21, R39, R56 and several mainchain nitrogen atoms. Notably, the key residues mediating both the sequence specific and DNA backbone interactions are conserved among EcmrR, BmrR and CueR (Supplementary Fig. 1a), consistent with the highly similar spacer DNA remodeling by the three MerR family regulators (Supplementary Fig. 7).

Although EcmrR and σ^70^ bind to opposite faces of the promoter, they engage in a direct interaction in the EcmrR-RP_o_ complex. The EcmrR NTD and R157–D160 short helix of σ^70^ NCR are in close proximity, as evidenced by their connected cryo-EM density, bury 228 Å^2^ of their surface area and make several favorable interactions through their surface residues (Fig. 2b and Supplementary Fig. 2). To investigate the effects of these contacting residues on EcmrR-mediated transcription activation, we constructed a σ^70^ R157A/D160A/K264A mutant (designated σ^70^ M1) and a series of EcmrR mutants, aiming to break the EcmrR NTD-σ^70^ NCR interaction. Almost all of the mutants, except EcmrR D35A, lead to significantly lower transcription activity on an EcmrR-dependent promoter (Fig. 2c,d). The greatly reduced transcription activation by EcmrR is not likely due to the impairment of incorporation of σ^70^ M1 into the RNAP holoenzyme or promoter-binding by EcmrR mutants, as indicated by the in vitro RNAP holoenzyme reconstitution assay and the bacterial one hybrid system-based in vivo DNA-binding assay^22^ (Supplementary Fig. 8a–d). Taken together, these results suggest a critical and unexpected role of σ^70^ NCR in EcmrR-mediated transcription activation.

Interestingly, in addition to its role in stabilizing EcmrR binding, σ^70^ NCR R157 was also shown to facilitate template DNA unwinding by directly interacting with the spacer region of a canonical promoter containing a 17-bp spacer^23^. Therefore, through two distinct modes of action, R157 contributes to open complex formation during the transcription initiation on both canonical and EcmrR-dependent promoters.

To further dissect the role of EcmrR NTD-σ^70^ NCR interaction in EcmrR-mediated transcription activation, we examined the transcription activity on a promoter containing EcmrR-binding sequence and a pre-melted bubble. While both WT σ^70^ and σ^70^ M1 support robust transcription in the absence of EcmrR, supplying the reaction with EcmrR protein does not further increase the transcription activity, suggesting EcmrR primarily functions in the open complex formation stage (Supplementary Fig. 8e, f). The slightly lower transcription activity accompanying σ^70^ M1 compared with WT σ^70^ implies that some or all of the three mutated residues (R157, D160 and K264) in σ^70^ NCR may play a minor role in stabilizing the RNAP-DNA complex formed on the 19-bp spacer promoter via an EcmrR-independent mechanism.

### Initial transcribing complexes

**Fig. 3:**
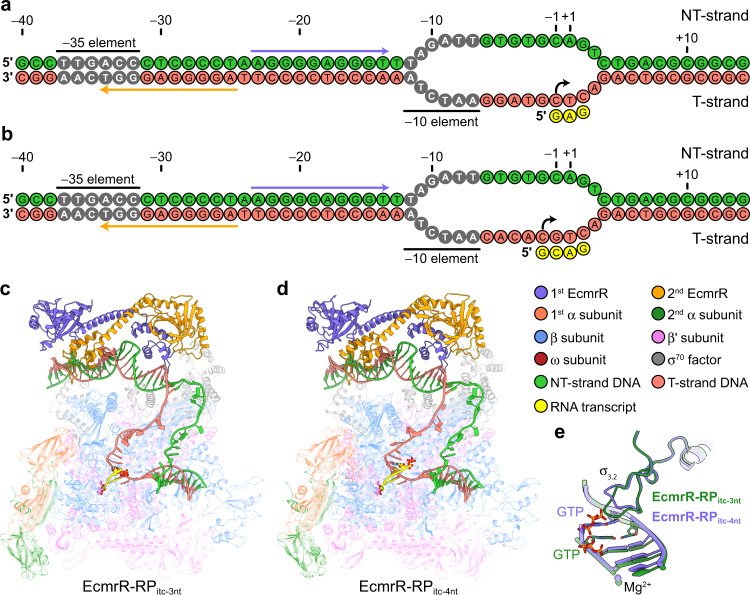
Structures of EcmrR-RNAP-promoter initial transcribing complexes. **a** Schematics of synthetic promoter DNA scaffolds and de novo synthesized RNA transcripts in EcmrR-RP_itc-3nt._ The two halves of the palindromic sequence recognized by EcmrR dimer are highlighted by medium blue and orange arrows, respectively. The transcription start site is indicated by a curved arrow. NT-strand, non-template strand; T-strand, template strand. **b** Schematics of synthetic promoter DNA scaffolds and de novo synthesized RNA transcripts in EcmrR-RP_itc-4nt_. **c** Overall cryo-EM structure of EcmrR-RP_itc-3nt_. The 5′-end GTP in the nascent RNA transcript and three catalytic aspartate residues (β' D460, β' D462 and β' D464) in the polymerase active center are shown as sticks. The Mg^2+^ ions are shown as green spheres. **d** Overall cryo-EM structure of EcmrR-RP_itc-4nt_. **e** Superimposition of RNA-DNA hybrid duplex and σ_3.2_ loop in EcmrR-RP_itc-3nt_ (forest green) and EcmrR-RP_itc-4nt_ (medium blue) illustrates the slight uplift of σ_3.2_ loop to avoid steric clashes when the length of RNA transcript reaches 4 nt.

The reconstituted RNAP-promoter-EcmrR complex is active for RNA synthesis under the cryo-EM buffer conditions. When including ribonucleoside triphosphates (rNTPs) in the complexes (see METHOD DETAILS), we obtained two structures of the RNAP-promoter initial transcribing complexes (RP_itc_) containing either a 3-nt de novo synthesized RNA transcript on the heteroduplex DNA scaffold (hereinafter designated EcmrR-RP_itc-3nt_) or a 4-nt RNA transcript on a fully complementary DNA scaffold (hereinafter designated EcmrR-RP_itc-4nt_) (Fig. 3a–d). The RNA transcripts lie at the post-translocation position in both structures and RNA synthesis starts at position –1 and –2 in EcmrR-RP_itc-3nt_ and EcmrR-RP_itc-4nt_, respectively (Fig. 3a, b). The selection of transcription start site (TSS) in each structure is supported by the initiating guanosine-5’-triphosphate (GTP) in nascent RNA strands and a T-strand DNA purine that is immediately upstream of and stacks with the RNA 5’ GTP, both of which are preferred features for TSS selection^24^.

Compared with EcmrR-RP_o_, both EcmrR-RP_itc-3nt_ and EcmrR-RP_itc-4nt_ unwind the T:A base pair at position +3 and pull it into the polymerase through DNA scrunching^25^, leading to a 1-nt transcription bubble expansion (Figs. 1c and 3a, b). Despite the same 14-nt transcription bubble size in both EcmrR-RP_itc_ structures, only the longer RNA transcript in EcmrR-RP_itc-4nt_ reaches and slightly lifts up the σ_3.2_ loop (Fig. 3e), indicating accumulation of significant stress on σ as the RNA transcript extends beyond this length. Charge repulsion between the acidic tip of σ_3.2_ loop and 5′ triphosphate of the 3-nt or 4-nt RNA is mitigated by a Mg^2+^ ion, coordinated by the triphosphate oxygens and imine N7 atom of GTP (Supplementary Fig. 9a). A bridging interaction between the 5′ triphosphate and acidic σ_3.2_ loop was previously suggested^19^ but is not evident in our structures.

Other than the differences in transcription bubble size and σ_3.2_ loop conformation, the overall structures of EcmrR-RP_o_ and EcmrR-RP_itc-4nt_ are almost identical (Supplementary Fig. 9b). The kinked spacer DNA conformation and all of the essential interactions between EcmrR, spacer DNA and σ^70^ NCR are maintained, suggesting that EcmrR-induced promoter remodeling is required not only for open complex formation, but also for initial transcription.

### EcmrR has two ligand-binding sites

**Fig. 4:**
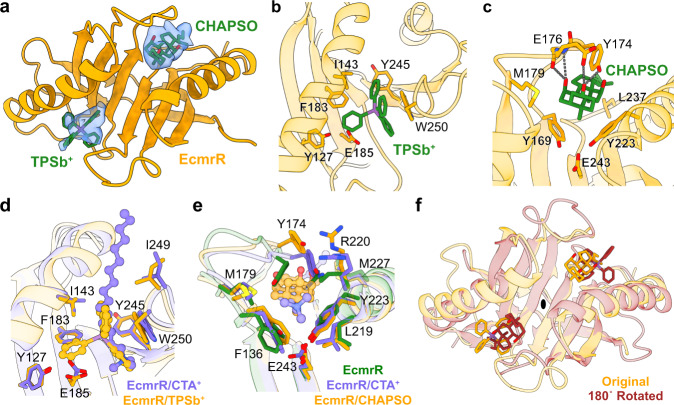
Two ligand-binding sites in EcmrR. **a** Overview of two ligand-bindings sites in EcmrR. Only the CTD of one EcmrR subunit is shown. Two bound ligands, TPSb^+^ and CHAPSO, are displayed as green sticks and are superimposed with the cryo-EM density (blue surfaces) contoured at 7σ. **b** Interactions between TPSb^+^ and EcmrR in ligand-binding site I. Ligand-interacting residues in EcmrR are shown as orange sticks. **c** Interactions between CHAPSO and EcmrR in ligand-binding site II. Ligand-interacting residues in EcmrR are shown as orange sticks. Hydrogen bonds are shown as gray dotted lines. **d** Comparison of EcmrR ligand-binding site I between the CTA^+^-bound and TPSb^+^-bound forms. Crystal structure of EcmrR CTD/CTA^+^ complex is superimposed with the structure of EcmrR/TPSb^+^/CHAPSO subcomplex in EcmrR-RP_itc-4nt_. Ligands are shown in ball-and-stick representation and EcmrR residues contacted by ligands are shown in stick representation. **e** Comparison of EcmrR ligand-binding site II among the unbound, CTA^+^-bound and CHAPSO-bound forms. Crystal structure of EcmrR CTD/CTA^+^ complex and EcmrR/TPSb^+^ subcomplex in EcmrR-RP_itc-3nt_ are superimposed with the structure of EcmrR/TPSb^+^/CHAPSO subcomplex in EcmrR-RP_itc-4nt_. **f** Pseudo two-fold symmetry of EcmrR CTD. The structure of EcmrR CTD with TPSb^+^ and CHAPSO bound to its two ligand-binding sites is rotated 180° along a hypothetical axis (indicated by a black ellipse) and overlapped with the original structure.

In the above three EcmrR-RNAP-promoter complexes, TPSb^+^ binds to a hydrophobic pocket (hereinafter designated site I) in EcmrR CTD (Fig. 4a, b). The location of EcmrR site I is close to but different from the multidrug-binding site in BmrR (Supplementary Fig. 10a). Intriguingly, we observed strong extra density in another hydrophobic pocket (hereinafter designated site II) in the cryo-EM maps of EcmrR-RP_o_ and EcmrR-RP_itc-4nt_ (Fig. 4a, c). The density likely corresponds to the tetracyclic sterol head of CHAPSO, which is routinely used in cryo-EM sample preparation of bacterial RNAP complexes to alleviate preferred orientation problems^21,26,27^. Dual site-binding is also observed in the 1.4 Å EcmrR CTD crystal structure, wherein both sites are occupied by cetyltrimethylammonium cation (CTA^+^) (Supplementary Fig. 10b), a cationic detergent in the crystallization solution. The overall shape and pocket residue conformations of site I remain largely unchanged between the CTA^+^- and TPSb^+^-bound forms except for a slight retraction of the E185 side chain to avoid clashes with one of the phenyl rings in TPSb^+^ (Fig. 4d). In comparison, site II is relatively flexible and undergoes significant conformational changes between its un-liganded, CTA^+^-bound and CHAPSO-bound forms (Fig. 4e). Y174 and R220, which partially occupy site II in the absence of ligand, gradually lift up as a ligand binds and as the bound ligand gets bulkier. The two ligand-binding sites in EcmrR are approximately symmetric about a pseudo-two-fold axis of the triangle-shaped EcmrR CTD (Fig. 4f and Supplementary Fig. 1e). By contrast, the single multidrug-binding site in BmrR CTD is roughly positioned on the axis (Supplementary Fig. 10a). Despite the differences in ligand-binding site locations and ligand-binding modes between EcmrR and BmrR, their ligand-binding pockets exhibit similar stereochemical properties.

Both ligand-binding pockets in EcmrR are surrounded by numerous aromatic and aliphatic amino acids that bury the hydrophobic ligands (Fig. 4b, c). A glutamate residue in each site (E185 and E243) dictates the preference for cationic compounds, while the lack of a positive charge in the tetracyclic sterol head of CHAPSO is compensated for by hydrogen bonds between its hydroxyl groups and polar residues on the surface of site II and extensive hydrophobic interactions in the ligand-binding pocket (Fig. 4b, c).

Unlike site I, which is occupied by TPSb^+^ in all of the three EcmrR-RNAP-promoter complexes, site II in EcmrR-RP_itc-3nt_ is vacant when CHAPSO was omitted during sample preparation, indicating that site II might have different selectivity for ligands compared with site I and that its occupancy is not essential for EcmrR-mediated transcription activation. Mutation of residues in site II leads to a mild, but still significant, decrease in transcription activation by EcmrR (Supplementary Fig. 10c). However, we cannot conclude from our existing data that the ligand-binding site II observed in our structures represent a biologically relevant drug-binding site that contribute to allosteric conformational change of EcmrR. Future studies examining the ligand-binding affinity and specificity are needed to delineate the role of site II in EcmrR-regulated transcription.

### Promoter escape and elongation complexes

**Fig. 5:**
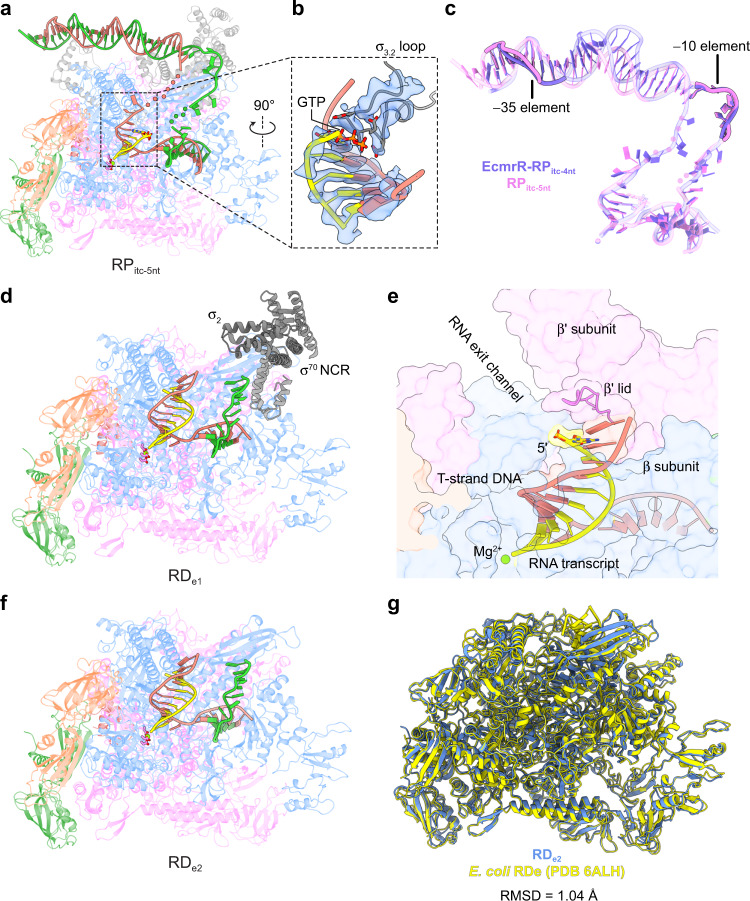
Promoter escape and elongation complex formation during EcmrR-dependent transcription. **a** Overall cryo-EM structure of RP_itc-5nt_. The color schemes for Fig. 5a, b, d–f are the same as in Fig. 3. The 5’-end GTP in the nascent RNA transcript and three catalytic aspartate residues (β' D460, β' D462 and β' D464) in the polymerase active center are shown as sticks. The Mg^2+^ ions are shown as green spheres. Disordered nucleotides in the transcription bubble region are indicated as salmon and green circles for T-strand and NT-strand, respectively. **b** A close-up view of the 5-nt RNA transcript and σ_3.2_ loop. The RNA 5’-end GTP and residues in the σ_3.2_ loop acidic tip are shown as sticks. The σ_3.2_ loop and RNA-DNA duplex are superimposed with their cryo-EM densities (blue surfaces) contoured at 4σ. **c** Comparison of the promoter DNA conformations in EcmrR-RP_itc-4nt_ and RP_itc-5nt_. Disordered nucleotides in the transcription bubble region of RP_itc-5nt_ are indicated as violet filled circles. The −10 elements on NT-strands and −35 elements on T-strands in the two structures are highlighted and labeled. **d** Overall cryo-EM structure of RD_e1_. **e** The 5’-end nucleotide (shown as sticks) of the 9-nt RNA transcript in RD_e_ structures reaches β' lid and is positioned at the entrance of RNA exit channel. **f** Overall cryo-EM structure of RD_e2_. **g** Superimposition of RD_e2_ with previously determined *E. coli* RD_e_ structure (PDB 6ALH).

Three additional structures containing RNA transcripts longer than 4-nt were determined from the complex reconstituted with a full set of rNTPs (Supplementary Fig. 4). As nascent RNA extends from 4 nt to 5 nt in the RNAP-promoter complex (hereinafter referred to as RP_itc-5nt_) (Fig. 5a, b), both the NT-strand and T-strand DNA in the bubble region become disordered compared with EcmrR-RP_itc-4nt_, likely due to DNA scrunching and stress buildup in the transcription bubble^25,28^ (Fig. 5a, c). In addition, the upstream edge of the pentanucleotide RNA transcript stacks tightly against σ_3.2_ loop (Fig. 5b). The stress-induced conformational change in the σ_3.2_ loop likely has been transmitted allosterically to the promoter-EcmrR binding interface and led to EcmrR dissociation (Fig. 5a). This in turn releases the kink and under-twist in promoter spacer DNA, which relaxes to adopt a B-form-like DNA structure (Fig. 5a, c, Supplementary Fig. 6a and Supplementary Video 1). Whereas the specific interactions between the –10 element and σ_2_ domain are maintained, the conformational change in spacer DNA extends the linear distance between –10 and –35 elements and rotates the –35 element out of the angular range for σ_4_ domain interaction (Fig. 5c). Although the HTH motif of σ_4_ domain still inserts into the DNA major groove, the 2-bp register shift significantly weakens its interaction with the –35 element, which likely contributes to increased promoter flexibility and less well-resolved cryo-EM density in this region (Supplementary Fig. 4). We propose that for transcription initiated from the EcmrR-dependent promoter, EcmrR dissociation and the accompanying promoter spacer relaxation demarcate the beginning of the promoter escape process.

When the RNA transcript length reaches 9 nt, σ_3.2_ loop is removed from the RNA elongation path and the RNA exit channel is cleared (Fig. 5d, e). The 5′ end of the 9-nt RNA transcript is at the entrance of the RNA exit channel and ready to be separated from the T-strand DNA by β' lid^29,30^ as RNA synthesis continues (Fig. 5e). We obtained two RNAP-DNA complex structures with 9-nt RNA transcripts, both of which appear to have undergone promoter escape, as they have no visible density for σ_3_ domain, σ_4_ domain, σ_3.2_ loop or upstream promoter DNA (Fig. 5d, f and Supplementary Fig. 4). Their overall structures resemble previously characterized RNAP-DNA elongation complexes (RD_e_)^30–32^ (Fig. 5g). However, one of the complexes (hereinafter referred to as RD_e1_, with the other elongation complex referred to as RD_e2_) still exhibits well-resolved density for σ^70^ NCR and σ_2_ domain (Fig. 5d and Supplementary Fig. 4), which have not previously been observed in bacterial RD_e_ structures. The observation of two distinct RD_e_ forms demonstrates stochastic release of σ^70^ from RNAP during elongation, as indicated by previous biochemical and biophysical studies^33–37^.

**Fig. 6:**
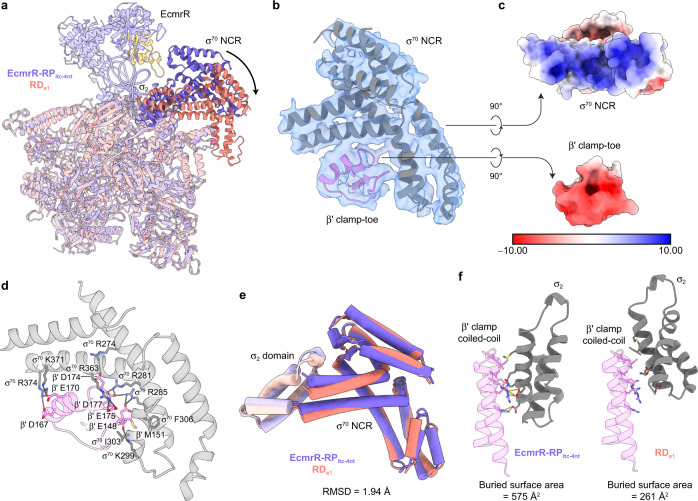
σ^70^ NCR adopts a different conformation and interacts with β' clamp during promoter escape. **a** Superimposition of RD_e1_ with EcmrR-RP_itc-4nt_ structures illustrates the large conformational changes of σ^70^ NCR and σ_2_ domain. The EcmrR NTD that is proximal to σ^70^ NCR is colored in yellow. **b** A close-up view of the σ^70^ NCR and β' clamp-toe interface superimposed with the cryo-EM density (blue surface) contoured at 5.5 σ. **c** Electrostatic surfaces of σ^70^ NCR and β' clamp-toe. The unit of electrostatic potential is kT/e^−^. **d** Interactions between σ^70^ NCR and β' clamp-toe. Hydrogen bonds and salt bridges are shown as gray dotted lines. **e** Superimposition of σ^70^ NCR (bright colors) together with σ_2_ domain (pale colors) in RD_e1_ with those in EcmrR-RP_itc-4nt_, showing that σ_2_ domain stays largely static relative to σ^70^ NCR in the two structures. **f** Comparison of the σ_2_-β' clamp coiled-coil interactions between the structures of EcmrR-RP_itc-4nt_ and RD_e1_. Hydrogen bonds and salt bridges are shown as yellow dotted lines. The surface areas buried between β' clamp coiled-coil and σ_2_ in the two structures are indicated.

With σ^70^ still bound to core RNAP, RD_e1_ provides a unique opportunity to understand the role of σ factor in promoter clearance. Compared with its position in RP_itc_, σ^70^ NCR in RD_e1_ has undergone a major 39° rotation and 21 Å centroid movement (Fig. 6a and Supplementary Video 1). It straddles a globular protrusion (V145–K179) in β' clamp (also known as β' clamp-toe^38^) and binds to the acidic surface of this region with an array of basic residues R274, R281, R285, K299, R363, K371 and R374 (Fig. 6b–d). Such β' clamp-σ^70^ NCR interactions have not previously been observed in RP_o_, RP_itc_ or RD_e_ structures.

Although residues in the σ^70^ NCR basic patch were suggested to play a role in promoter escape^39^, it is not known how they interact with RNAP core enzyme to facilitate this process. Our structures reveal that σ_2_ domain stays largely static relative to σ^70^ NCR from RP_itc_ to RD_e1_ (Fig. 6e). The major rotation of σ^70^ NCR is concomitant with a significant repositioning of σ_2_ domain in RNAP and likely abolishes its interactions with β' clamp coiled-coil (Fig. 6a, f and Supplementary Video 1), which was shown to be important for early elongation pause and impede promote escape^39,40^, and with the –10 promoter element. As σ^70^-promoter interactions at both the –35 and –10 elements are lost at this point, the upstream edge of the transcription bubble is free to move during processive transcription elongation.

## Discussion

**Fig. 7:**
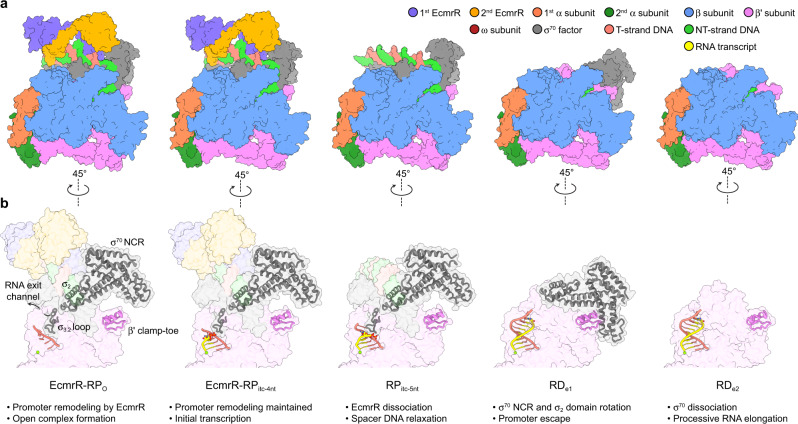
Structural visualization of EcmrR-dependent transcription. **a** Side-by-side comparison of five cryo-EM structures captured at different stages during EcmrR-dependent transcription. **b** σ_3.2_ loop, σ_2_ domain and NCR of σ^70^, β' clamp-toe and RNA-DNA duplex are shown as cartoons, while other parts of the complexes are shown as transparent surfaces. For clarity, promoter DNA downstream of the –10 element, RNAP α and β subunits are not shown.

The series of structures described here capture multiple steps in multidrug-activated transcription (Fig. 7a, b) and provide mechanistic insights into the regulation of the transcription initiation by the unique MerR family factors. As with all other members of the MerR family, spacer DNA remodeling is an essential step in EcmrR-mediated transcription activation. Our structures reveal how EcmrR remodels the suboptimal promoter through bending and under-twisting the 19-bp spacer to achieve optimal promoter conformation for RNAP recognition in response to multidrug binding during both open complex formation and initial transcription. In addition, our structures show that promoter reconfiguration by EcmrR is aided by a previously unknown interaction with σ^70^ NCR, although further investigations are needed to determine the extent to which this EcmrR-σ^70^ interaction contributes to EcmrR-dependent transcription activation. More importantly, as both of the EcmrR-DNA and EcmrR-σ^70^ interactions are mediated by the EcmrR NTD, a well-defined DNA-binding domain with conserved sequence and structure throughout the MerR family regulators^6^, it is plausible that the mechanism of transcription activation by EcmrR is also adopted by other members of the MerR family.

The structure of RP_itc-5nt_ sheds first light on the time point of EcmrR release from the promoter following transcription activation (Fig. 7a, b). As the growing RNA transcript reaches 5 nt in length, the stress built up in σ factor and promoter upstream DNA eventually leads to the disruption of the EcmrR-σ^70^ and EcmrR-promoter interactions. The dissociation of EcmrR from the RNAP-promoter complex reverses promoter DNA remodeling, breaks the −35 element recognition and frees σ^70^ NCR to establish previously unobserved interactions with RNAP β' clamp during promoter escape, which is visualized in our RD_e1_ structure (Fig. 7a, b).

In the RD_e1_ structure, σ^70^ NCR would be too far apart from EcmrR to form interactions if the latter was to be present in the structure (Fig. 6a), implying the interactions of σ^70^ NCR with EcmrR NTD or β' clamp are mutually exclusive. Therefore, the binding and release of EcmrR, and likely other MerR family regulators, from the RNAP-promoter complex are accompanied by the switch between these two distinct functional modes of σ^70^ NCR. The concerted actions of MerR family regulators and σ^70^ NCR enable them to achieve functionally distinct outcomes during the dynamic processes of promoter recognition and promoter escape.

Notably, the σ^70^ NCR residues involved in the interactions with either EcmrR or β' clamp have also been shown to be important for the open complex formation^23^ and promoter escape^39^ stages of transcription from promoters with canonical 17-bp spacers. Thus, the dual functional modes of σ^70^ NCR revealed by our structures could be generally applicable to most, if not all, σ^70^-initiated transcription processes, rather than being limited to the MerR family regulators-dependent transcription.

## Methods

### Cloning of EcmrR

EcmrR (GenBank accession number: WP_089517995.1) is annotated as an MerR family transcriptional regulator. Its homologs are widely present in various species in the Enterobacterales order and share higher than 90% sequence identity with EcmrR. The gene encoding EcmrR was codon-optimized and synthesized from GenScript. The full length or C-terminal domain (CTD, residues 112–269) of EcmrR fused to an N-terminal His_6_-Smt3 was cloned into the pETDuet^TM^-1 expression vector (MilliporeSigma) between NcoI and HindIII restrictive sites. EcmrR containing point mutations were constructed using site-directed mutagenesis. Mutations were confirmed by sequencing. The sequences of all nucleotide oligos used in this study are listed in Supplementary Table 2.

### Protein expression and purification

His_6_-Smt3 tagged EcmrR proteins were overexpressed in *E. coli* BL21 Star (DE3) (ThermoFisher Scientific) at 17 °C for 16 h. Cells expressing His_6_-Smt3 tagged EcmrR CTD fragment were re-suspended in buffer A (50 mM Tris-HCl, pH 7.5, 200 mM NaCl, 5% glycerol, 1 mM β-mercaptoethanol (β-ME), 20 mM imidazole) and lysed by six passes through an M110EH Microfluidizer (Microfluidics International Corporation). Cell lysate was cleared by centrifugation at 34,572 *g* using a SS-34 rotor (ThermoFisher Scientific) for 1 h at 4 °C. The supernatant was loaded onto a HisTrap HP affinity chromatography column (GE Healthcare), washed with 5× column volume (CV) of buffer A and eluted with a linear gradient from buffer A to buffer B (50 mM Tris-HCl, pH 7.5, 200 mM NaCl, 5% glycerol, 1 mM β-ME, 300 mM imidazole) over 20× CV. Fractions containing target protein were pooled and diluted with equal volume of buffer C (20 mM Tris-HCl, pH 8.0, 2 mM β-ME). The His_6_-Smt3 tag was removed by adding 2 mg of Ulp1 protease to target protein solution and incubating at 4 °C for 16 h. The sample was then loaded onto a HiTrap Q HP anion exchange chromatography column (GE Healthcare), washed with 5× CV of buffer C and eluted with a linear gradient from buffer C to buffer D (20 mM Tris-HCl, pH 8.0, 1 M NaCl, 2 mM β-ME) over 20× CV. Fractions corresponding to tag-free EcmrR CTD were pooled, further purified and buffer exchanged using a HiLoad 26/60 Superdex 200 pg size-exclusion chromatography (SEC) column in buffer E (20 mM Tris-HCl, pH 7.5, 100 mM NaCl, 1 mM dithiothreitol (DTT)).

Cells expressing His_6_-Smt3 tagged full-length EcmrR were re-suspended in buffer F (20 mM Tris-HCl, pH 7.5, 500 mM KCl, 5% glycerol, 20 mM imidazole) and lysed by six passes through an Emulsiflex C3 homogenizer (Avestin). Cell lysate was cleared by centrifugation at 191,402 *g* using a Type 50.2 Ti rotor (Beckman Coulter) for 1 h at 4 °C and was mixed with pre-equilibrated Ni-NTA Agarose resin (Qiagen) for 1 h with continual rotation. The resin was loaded into a gravity flow column, washed with 5× CV of buffer G (20 mM Tris-HCl, pH 7.5, 1 M KCl, 5% glycerol, 20 mM imidazole). The target protein was eluted with 5× CV of buffer H (20 mM Tris-HCl, pH 7.5, 500 mM KCl, 5% glycerol, 150 mM imidazole), followed by purification using a Superdex 200 Increase 10/300 GL SEC column (GE Healthcare) in buffer I (20 mM Tris-Cl, pH 7.5, 500 mM KCl, 1 mM Tris(2-carboxyethyl) phosphine hydrochloride (TCEP-HCl)).

*E. coli* core RNAP was expressed in *E. coli* BL21 (DE3) (ThermoFisher Scientific), and purified using HisTrap HP affinity chromatography column (GE Healthcare), followed by HiTrap Heparin HP column (GE Healthcare), HiTrap Q HP anion exchange chromatography column (GE Healthcare) and HiLoad 26/60 Superdex 200 pg size-exclusion chromatography (SEC) column. To express *E. coli* σ^70^ protein, the coding fragment was cloned into pET21a plasmid between *Nhe* I and *Hind* III sites to produce pET21a-EcorpoD. Mutations in σ^70^ were constructed using site-directed mutagenesis based on the pET21a-EcorpoD plasmid. Wild-type and mutated σ^70^ were expressed in *E. coli* BL21 (DE3) and purified using HisTrap HP affinity chromatography column (GE Healthcare), followed by HiTrap Heparin HP column (GE Healthcare) and HiLoad 26/60 Superdex 200 pg size-exclusion chromatography (SEC) column.

### Crystallization and data collection

Purified tag-free EcmrR CTD was concentrated to 20 mg/ml and used in crystallization screening. EcmrR CTD crystals were grown by sitting-drop vapor diffusion at 19 °C in 0.35–0.5 M NaCl, 10 mM MgCl_2_, 5 mM cetyltrimethylammonium bromide (CTA^+^•Br^–^). Crystals were grown for 2 weeks and cryoprotected in 0.6 M NaCl, 10 mM MgCl_2_, 5 mM CTAB, 35% (v/v) glycerol and flash-frozen in liquid nitrogen. Heavy atom derivatives of EcmrR CTD were prepared by soaking the crystals in 1 M NaBr, 10 mM MgCl_2_, 5 mM CTAB, 35% (v/v) glycerol, or 0.5 M NaCl, 0.5 M NaI, 10 mM MgCl_2_, 5 mM CTAB, 35% (v/v) glycerol for 2–5 min. X-ray diffraction data were collected at 100 K at beamline 24ID-C of the Advanced Photon Source at Argonne National Laboratory. The datasets for EcmrR CTD native, Br- and I-derivative crystals were collected at 0.9792 Å, 0.9197 Å and 1.4586 Å, respectively. All X-ray diffraction data were indexed, integrated and scaled using the XDS package^41^. The statistics of data collection are summarized in Supplementary Table 1.

### Crystal structure determination and refinement

Heavy atom sites were identified using SHELXD^42^. The structure was determined using one native crystal, one Br-derivative crystal and one I-derivative crystal by multiple isomorphous replacement with anomalous scattering (MIRAS) method in AutoSol^43^, and the initial model was built automatically using AutoBuild^44^. The model was manually rebuilt in Coot^45^ and refined in PHENIX^46^ with anisotropic B-factor for all non-hydrogen and non-water atoms. The final structure was refined to 1.4 Å with *R*_work_ and *R*_free_ of 0.1434 and 0.1734, respectively. All residues in the EcmrR CTD were built in the final model. Structure validation was carried with MolProbity^47^. 98.56% of the residues are in the favored regions of the Ramachandran plot, 1.44% in allowed regions, and none is the disallowed region.

### EcmrR-promoter-RNAP complex assembly

54-bp heteroduplex or fully complementary promoter DNA scaffolds were generated by annealing equimolar amounts of the two oligonucleotides (Integrated DNA Technologies, Inc.) shown in Fig. 1c and Fig. 3b, respectively. The sequence of the 19-bp spacer was adapted from the spacer DNA used in the structural studies of BmrR-DNA complexes (PDB 1EXI, 1EXJ, 3D6Z and 3Q3D). Both promoter DNA scaffolds were purified using a HiLoad 26/60 Superdex 200 pg SEC column in buffer J (20 mM Tris-HCl, pH 7.5, 100 mM KCl, 1 mM TCEP) and were then concentrated to 4 mg/ml.

Tag-free full-length EcmrR is highly unstable and readily precipitates. Therefore, EcmrR-promoter DNA subcomplexes were reconstituted before His_6_-Smt3 tag removal. His_6_-Smt3-tagged full-length EcmrR was mixed with either 54-bp heteroduplex or fully complementary promoter DNA in a 1:1.5 molar ratio at 4 °C for 30 min with continual rotation. The protein-DNA mixture was then buffer exchanged to 20 mM Tris-HCl, pH 7.5, 100 mM KCl, 1 mM TCEP in an Amicon Ultra centrifugal filters with 10 kDa molecular weight cutoff (MilliporeSigma), supplemented with 0.5 mg of Ulp1 protease and incubated at 4 °C for 16 h for tag removal. Tag-free EcmrR-promoter DNA subcomplexes were purified using a HiLoad 26/60 Superdex 200 pg SEC column in 20 mM Tris-HCl, pH 7.5, 100 mM KCl, 1 mM TCEP.

To reconstitute EcmrR-promoter-RNAP complex, *E. coli* core RNAP was mixed with σ^70^ and EcmrR-promoter subcomplex in a 1:1.4:2 molar ratio. The mixture was supplemented with 5 mM MgCl_2_ and 5 mM TPSb^+^•Br^–^ (Santa Cruz Biotechnology) and incubated at 23 °C for 3 h. For EcmrR-promoter-RNAP complex with a heteroduplex DNA scaffold, the mixture was either directly loaded onto a Superdex 200 increase 10/300 GL SEC column for EcmrR-RP_o_ complex purification or supplemented with 200 µM adenosine-5′-triphosphate (ATP) and 200 µM GTP, incubated at 37 °C for 10 min then loaded onto a Superdex 200 increase 10/300 GL SEC column for EcmrR-RP_itc-3nt_ complex purification. For EcmrR-promoter-RNAP complex with a fully complementary DNA scaffold, the mixture was supplemented with 200 µM GTP, 200 µM ATP, 200 µM cytidine-5′-triphosphate (CTP) and 2 µM uridine-5′-triphosphate (UTP), incubated at 37 °C for 10 min and purified using a Superdex 200 increase 10/300 GL SEC column.

### Cryo-EM sample preparation and data acquisition

Purified EcmrR-RP_itc-3nt_ complex (3.5 µl at ~1.2 µM) was applied to freshly glow-discharged Quantifoil 300 mesh R1.2/1.3 copper grids with holey carbon foil (Electron Microscopy Sciences). Grids were blotted for 3 s at 4 °C under 100% humidity and plunge-frozen in liquid nitrogen-cooled liquid ethane using a Vitrobot Mark IV (ThermoFisher Scientific). Cryo-EM data were collected using EPU version 2.5 (ThermoFisher Scientific) on a Titan Krios electron microscope (ThermoFisher Scientific) equipped with a Falcon III direct electron detector (ThermoFisher Scientific) at the Hormel Institute. The movies were collected at a nominal magnification of 96,000×, corresponding to 0.895 Å per pixel, at a dose rate of 0.8 e^–^ per pixel per second with a defocus ranging from –0.8 to –2.6 µm. The total exposure time for each movie is 30 s, leading to a total accumulated dose of 30 e^–^ Å^–2^, which was fractionated into 30 frames.

Other EcmrR-promoter-RNAP complexes were concentrated to ~12 µM and supplemented with 300 mM CHAPSO stock solution to a final concentration of 8 mM CHAPSO immediately before grid preparation. 3.5 µl of each complex was applied to freshly glow-discharged Quantifoil 300 mesh R1.2/1.3 gold grids with holey carbon foil (Electron Microscopy Sciences). Grids were blotted for 5 s at 22 °C under 100% relative humidity and plunge-frozen in liquid ethane. Cryo-EM datasets were collected using SerialEM version 3.6^48^ on a Titan Krios G2 electron microscope equipped with a K2 summit direct electron detector (Gatan, Inc.) in super-resolution mode at Yale Cryo-EM facility. The movies were collected at a nominal magnification of 105,000×, corresponding to 0.6665 Å per super-resolution pixel, at a dose rate of 7.5 or 6.8 e^–^ per physical pixel per second with a defocus ranging from –1.2 to –2.2 µm. The total exposure time for each movie is 12 s or 13.2 s, thus leading to a total accumulated dose of 50.68 or 50.46 e^–^ Å^–2^, which was fractionated into 40 or 44 frames, respectively. The statistics of cryo-EM data collection are summarized in Table 1.

### Image processing

Dose-fractionated movies were subjected to motion correction and dose-weighting using RELION’s own implementation of the UCSF MotionCor2 program^49^ in RELION-3.1^50^. For the datasets of EcmrR-RP_o_ and EcmrR-RP_itc-3nt_, the non-dose-weighted aligned images were used for contrast transfer function (CTF) with CTFFIND-4.1.13^51^. The dose-weighted images were used for classification and refinement in RELION-3.1. Particles were picked using the reference-free method of Gautomatch (https://www.mrc-lmb.cam.ac.uk/kzhang/). The autopicked particles were subjected to multiple rounds of 2D classifications to remove junk particles. Particles in good 2D classes were re-extracted for 3D classification. EMD-20090 map was rescaled to the same pixel and box sizes as the re-extracted particle stacks and low-pass filtered to 60 Å to serve as a starting reference for 3D classifications. Good 3D classes were selected and iteratively refined with C1 symmetry in RELION-3.1.

For the dataset collected using fully complementary promoter DNA scaffold, the dose-weighted aligned images were imported into cryoSPARC v2.14^52^ for patch-based CTF estimation, followed by Topaz micrograph denoise^53^ and Topaz particle-picking^54^. The picked particles were subjected to three rounds of 2D classification to remove junk particles. Particles in good 2D classes were selected for generation of multiple initial models that were low-pass filtered to 20 Å to serve as starting references for heterogeneous refinement (3D classification) in cryoSPARC v2.14. Particles in good 3D classes were selected and imported back into RELION-3.1 using csparc2star.py module^55^ for re-extraction and 3D auto-refinement with C1 symmetry.

All maps were further improved through iterative CTF refinement^50^ and Bayesian polishing^56^ in RELION-3.1. The final particle stacks were imported into cryoSPARC v2.14 again for non-uniform refinement^57^ to achieve the best resolution and map quality. To improve the map quality and interpretability of the EcmrR part in the EcmrR-promoter DNA-RNAP complexes, the final particle stacks were subjected to particle subtraction to keep only the signal of EcmrR dimer and the promoter spacer region, followed by masked local 3D refinement in RELION-3.1. To improve the map quality for σ^70^ NCR, particles in the final good 3D class(es) were subjected to masked 3D classification focusing on σ^70^ NCR with residual signal subtraction. The overall map resolution was estimated based on the Fourier shell correlation (FSC) cutoff at 0.143 between the two half-maps, after a soft mask was applied to mask out solvent region. The final maps were sharpened either automatically during 3D refinement or separately after refinement in cryoSPARC v2.14. Local resolution variation was estimated from the two half-maps in cryoSPARC v2.14. Histogram and directional FSC plots for the cryo-EM maps were analyzed and generated by 3DFSC web server^58^.

### Cryo-EM model building and refinement

Crystal structure of EcmrR CTD was fitted as rigid body into the EcmrR-promoter spacer complex map resulted from masked local 3D refinement. The atomic model of EcmrR NTD was built de novo into the 3.0 Å map. The structure of RP_o_ containing a 17-bp spacer promoter (PDB 6OUL) was first docked into the EcmrR-promoter-RNAP complex maps and then subjected to flexible fitting using ISOLDE^59^. The promoter DNA from PDB 6OUL was then mutated to the 54-bp promoter DNA scaffolds used in this study in Coot. Cryo-EM maps of RD_e1_ and RD_e2_ contain clear density for nine RNA nucleotides that are base-paired with T-strand DNA and additional weak density corresponding to single-stranded RNA in the RNA exit channel. However, the density feature is not good enough for unambiguous assignment of RNA register. This is likely because the particles used to reconstruct the RD_e_ maps are at different position on the template DNA and thus have variable length of RNA transcripts. When building the models of RD_e1_ and RD_e2_, only nine ribonucleotides were built in the maps with an initiating guanosine at the 5’ end. The 9-nt RNA transcripts are in the post-translocation position in both RD_e1_ and RD_e2_. The structural models of EcmrR and RNAP-promoter complex were merged in Coot and manually rebuilt and refined using PHENIX real space refinement^60^ with secondary structure restraints, rotamer restraints and Ramachandran restraints. The final structures were validated with MolProbity. The statistics of cryo-EM refinement were summarized in Table 1. Molecular representations were prepared using UCSF ChimeraX^61^. Structure-based sequence alignment of EcmrR, BmrR and CueR was performed using PROMALS3D web server^62^ and displayed using the Espript 3.0 web server^63^.

### Structural analysis of promoter spacer DNA

UCSF Chimera was used to measure the spacer lengths and kink angles. The spacer length is defined as the centroid-to-centroid distance between the first and last base pairs of a promoter spacer DNA. The kink angle is defined as the angle between the centroids of the first six base pairs, central seven base pairs and last six base pairs, respectively, of a 19-bp spacer (or the first five base pairs, central seven base pairs, and last five base pairs of a 17-bp spacer). Promoter spacer DNA conformation was analyzed by W3DNA 2.0 web server^64^. The total helical twist angles of the promoter spacers were calculated by extrapolating the average base-pair step helical twist (h-twist) parameter over 19 baes-pair steps. The under-twist value of each spacer DNA was calculated by subtracting its total helical twist angle from the total helical twist angle of an ideal B-form 19-bp DNA.

### In vitro transcription assay

In vitro transcription assays were performed in a KCl containing TB buffer (20 mM Tris-HCl, pH 7.9, 50 mM NaCl, 10 mM KCl, 5 mM MgSO_4_, 1 mM DTT, 0.1 mM EDTA, 5% glycerol). A 135-bp fully duplex DNA scaffold containing an EcmrR-dependent promoter was generated by first annealing two partially complementary oligonucleotides (5′-CCG GAT GCA AAT CGA GCC GAT TTT TTA ATC TTT ACG GAC TTT TAC CCG CCT GGT TTA TTA ATT TCT TGA CC-3′ and 5′-CCG CGC ACT CCT TTA AGA CAG TTT TGA CTG GCT GCA CAC AAT CTA AAC CCT CCC CTT AGG GGA GGG TCA AGA AAT TAA TAA ACC AGG-3′. The pseudo-palindromic sequence recognized by EcmrR is underlined) followed by DNA polymerase extension to full-length double-stranded DNA. 20 nM of DNA scaffold was incubated with different concentrations of wild-type or mutant EcmrR protein at 37 °C for 10 min. The complexes were then incubated with 100 nM pre-assembled σ^70^-RNAP holoenzyme at 37 °C for another 10 min. Transcription was initiated by the addition of 50 μM CTP, UTP and ATP, 5 μM GTP, and 1 μCi of [α-^32^P]GTP. The reactions were carried out at 37 °C for 10 min and stopped by 1 volume of 95% formamide solution. RNA products were incubated at 70 °C for 5 min and analyzed on denaturing 16% polyacrylamide gels. To form a DNA scaffold containing a pre-melted bubble, two DNA oligonucleotides (5′-GCC TTG ACC CTC CCC TAA GGG GAG GGT TTA GAT TGT ATG CTC AGT GTA TCC CGG GCG-3′ and 5′-CGC CCG GGA TAC ACA CTC GTA GGA ATC TAA ACC CTC CCC TTA GGG GAG GGT CAA GGC-3′) were annealed by heating to 95 °C and gradually cooling down to 15 °C. In vitro transcription was performed similarly as described above except that 50 μM GTP, UTP and ATP, 5 μM ATP, and 1 μCi of [α-^32^P]ATP were used as substrates and the transcripts were analyzed by 20% polyacrylamide gels. To test the effects of drug-like compounds on EcmrR-mediated transcription activation, the reaction mixtures, which contain 300 nM EcmrR, were supplemented with different concentrations of rhodamine 6 G (R6G) (Aladdin), kanamycin sulfate (BBI), tetraphenylphosphonium (TPP^+^) (Aladdin), or 3-([3-cholamidopropyl]dimethylammonio)-2-hydroxy-1-propanesulfonate (CHAPSO) (Aladdin) before the addition of rNTPs. RNA synthesis and detection were performed as described above.

### In vivo promoter activity test

In vivo promoter activity was tested using a low copy *lacZ* reporter fusion plasmid pZT100^65^. An EcmrR-dependent promoter fragment was cloned into pZT100 using ClonExpress II One Step Cloning Kit (Vazyme). DNA sequences encoding EcmrR with a C-terminal His_6_ tag were cloned into pBAD22 plasmid^66^. Mutations in the EcmrR coding region were introduced by QuickChange site-directed mutagenesis kit (Stratagene). The pZT100 and pBAD22 constructs were co-transformed into a *lacZ*-deleted *E. coli* strain^67^. Bacteria were grown at 37 °C to exponential phase in the presence of 0.2% L-arabinose. Promoter activities were tested by measuring β-galactosidase activities. The expression of EcmrR in the transformed *E. coli* strains was validated by western blot using anti-His antibody (ABBkine, 1:2000 dilution in phosphate-buffered saline (PBS) containing 0.5% Tween-20). The expression of RpoA protein, which was used as a loading control, was detected by anti-RpoA serum made in-house (1:1000 dilution in PBS containing 0.5% Tween-20). HRP-conjugated goat Anti-rabbit IgG (Beyotime, 1:10000 dilution in PBS) was used for chemiluminescence detection.

### In vivo DNA binding assay

In vivo DNA binding was tested using the bacterial one-hybrid system^22,68^. A DNA fragment (~300 bp) containing a weak *lac* promoter (designated P_control_) was cloned into the *lacZ* reporter fusion plasmid pZT100 to form the control plasmid. The EcmrR-recognizing sequence (designated EcmrR box) was introduced upstream of the weak promoter in P_control_ by QuickChange site-directed mutagenesis (Stratagene) to form P_EcmrR_. The sequence encoding either wild-type or mutant EcmrR proteins was cloned into a derivative of the pBRα plasmid^69^ to express EcmrR proteins fused to the C-terminus of RNAP α subunit (designated α-EcmrR). The pBRα derivates were co-transferred with P_control_ or P_EcmrR_ into a *lacZ*-deleted *E. coli* strain. *E. coli* cells were grown at 37 °C to exponential phase in the presence of 0.5 mM IPTG. Promoter activities were quantified by measuring β-galactosidase activities and were normalized relative to the activities in the *E. coli* strains without wild-type or mutant α-EcmrR fusion proteins.

### Quantification and statistical analysis

RNAs from in vitro transcription assays were quantified by ImageJ software^70^. In most cases, two distinct RNA bands are visible at the approximate position of the expected ~33-nt run-off transcript, likely due to the heterogeneity in transcription start site selection. Therefore, both bands were quantified and their sum was used for plotting. Data are shown as mean ± SD from three experiments. The β-galactosidase activity data were obtained from three colonies performed in duplicates for each strain and data are shown as mean ± SEM. Statistical analyses were performed using the unpaired Student’s *t*-test (two-tailed) between each of two groups.

### Reporting summary

Further information on experimental design is available in the Nature Research Reporting Summary linked to this paper.

## Supplementary information

Supplementary Information

Supplementary Movie 1

Description of Additional Supplementary Files

Reporting Summary

## Data Availability

Atomic coordinates of ten structures have been deposited in PDB with accession numbers 6WL5 (EcmrR CTD), 6XL5 (EcmrR-RP_o_), 6XL6 (EcmrR-spacer DNA complex from EcmrR-RP_o_), 6XL9 (EcmrR-RP_itc-3nt_), 6XLA (EcmrR-spacer DNA complex from EcmrR-RP_itc-3nt_), 6XLJ (EcmrR-RP_itc-4nt_), 6XLK (EcmrR-spacer DNA complex from EcmrR-RP_itc-4nt_), 6XLL (RP_itc-5nt_), 6XLM (RD_e1_) and 6XLN (RD_e2_). Ten cryo-EM density maps of different EcmrR and RNAP complexes have been deposited in the Electron Microscopy Data Bank with accession number EMD-22234 (EcmrR-RP_o_), EMD-23291 (EcmrR-RP_o_ with clear σ^70^ NCR-EcmrR NTD interface density), EMD-22235 (EcmrR-spacer DNA complex from EcmrR-RP_o_), EMD-22236 (EcmrR-RP_itc-3nt_), EMD-22237 (EcmrR-spacer DNA complex from EcmrR-RP_itc-3nt_), EMD-22245 (EcmrR-RP_itc-4nt_), EMD-22246 (EcmrR-spacer DNA complex from EcmrR-RP_itc-4nt_), EMD-22247 (RP_itc-5nt_), EMD-22248 (RD_e1_), EMD-22249 (RD_e2_), respectively. Source data are provided with this paper.

## Source data

Source Data
