# Supplementary material for: Structural visualization of transcription activated by a multidrug-sensing MerR family regulator
Source: Nat Commun. 2021 May 11;12:2702. doi: 10.1038/s41467-021-22990-8 (PMC8113463; doi:10.1038/s41467-021-22990-8)
Supplement: Supplementary file 1 — Supplementary Information [file 41467_2021_22990_MOESM1_ESM.pdf]

Supplementary Information for

Structural visualization of transcription activated by a multidrug-sensing MerR family regulator

Yang Yang†*, Chang Liu†*, Wei Zhou†, Wei Shi†, Ming Chen, Baoyue Zhang, David G. Schatz, Yangbo Hu* & Bin Liu*

†These authors contributed equally: Yang Yang, Chang Liu, Wei Zhou, and Wei Shi

*Correspondence: yan9yang@iastate.edu (Y.Y.), c.liu@yale.edu (C.L.), ybhu@wh.iov.cn (Y.H.), liu00794@umn.edu (B.L.)

Supplementary Figures 1-10

Supplementary Table 1,2

Supplementary Video 1

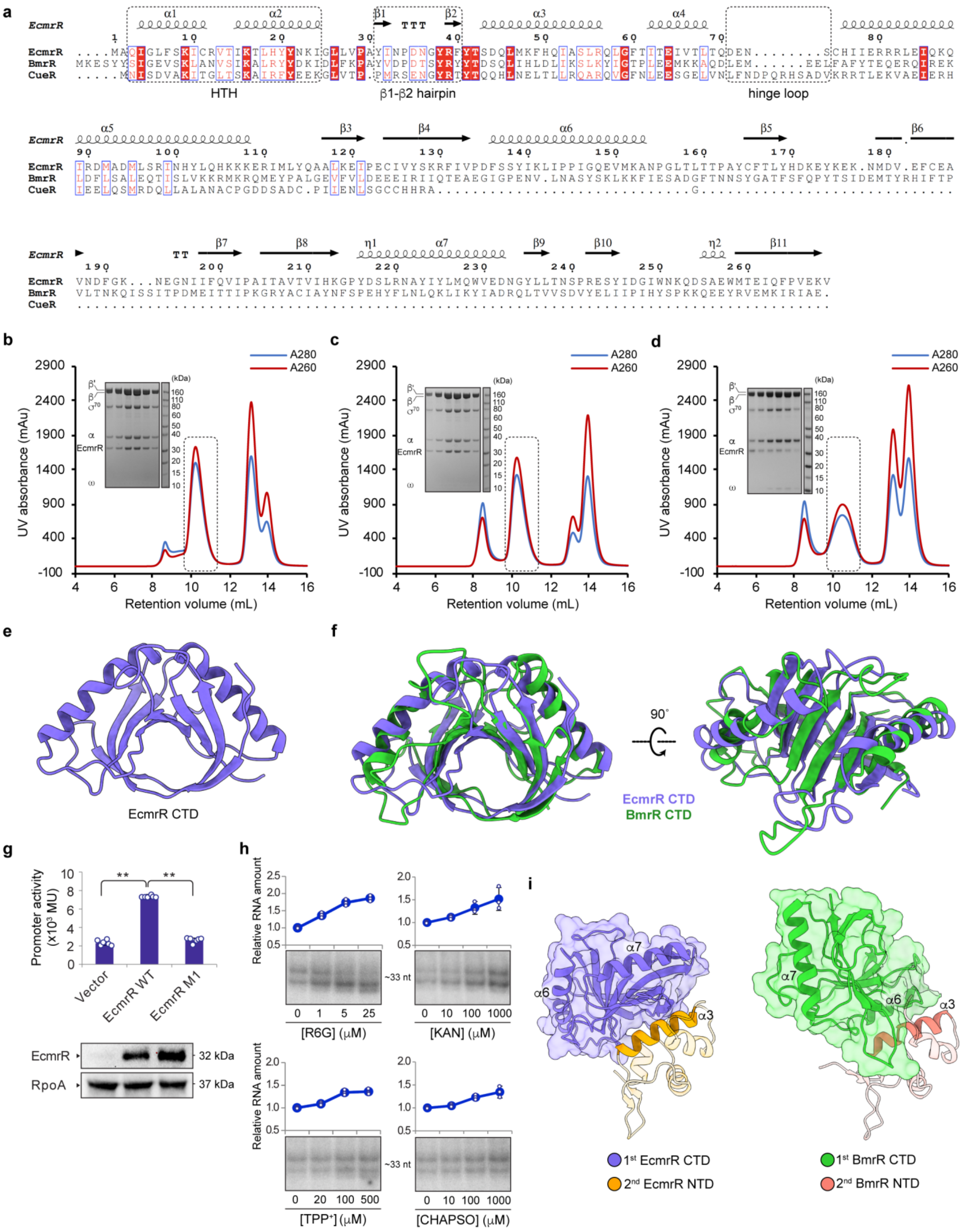

Supplementary Fig. 1: Structural and functional characterization of EcmrR.

a Sequence alignment of EcmrR, BmrR and CueR. Residue numbering and secondary structure annotations are for EcmrR. The DNA-binding Helix-turn-helix (HTH) motif, β1-β2 hairpin and hinge loop are boxed with dotted lines and labeled. **b** Size exclusion chromatography (SEC) profiles and SDS-PAGE analyses of EcmrR-RNAP-promoter complexes reconstituted using a heteroduplex DNA scaffold. The protein compositions in the dotted line boxed fractions from each complex reconstitution are shown in the embedded SDS-PAGE panels. A representative result from three independent experiments is shown. **c** Size exclusion chromatography (SEC) profiles and SDS-PAGE analyses of EcmrR-RNAP-promoter complexes reconstituted using a heteroduplex DNA scaffold with ATP and GTP. A representative result from three independent experiments is shown. **d** Size exclusion chromatography (SEC) profiles and SDS-PAGE analyses of EcmrR-RNAP-promoter complexes reconstituted using a fully complementary DNA scaffold with a full set of rNTPs. A representative result from three independent experiments is shown. **e** Crystal structure of EcmrR CTD. **f** Front and top views of EcmrR CTD superimposed with BmrR CTD (PDB 1EXI). **g** Activation of *in vivo* transcription from an EcmrR-dependent promoter by EcmrR WT and EcmrR M1. The promoter is fused to a β-galactosidase reporter gene (*lacZ*). Promoter activity was measured by β-galactosidase activity in miller units (MU). Expression levels of EcmrR and the loading control RpoA are shown. The results indicate that the decrease in transcription activation by EcmrR M1 is not due to large decrease in protein expression. Data were obtained from three colonies performed in duplicate and are shown as mean ± SEM. Statistical analyses were performed using the unpaired Student's *t*-test (two-tailed). **$P < 0.01$. **h** EcmrR-mediated transcription activation is further enhanced by various drug-like compounds. The concentrations (in μM) of each compound are indicated. R6G, rhodamine 6G; KAN, kanamycin; TPP^+, tetraphenylphosphonium; CHAPSO, 3-([3-cholamidopropyl]dimethylammonio)-2-hydroxy-1-propanesulfonate. The experiment was repeated three times and similar results were obtained. RNA products were quantified from these three independent experiments and are shown as mean ± SD. Both of the two distinct RNA bands

were quantified, and their signals are combined for plotting. Uncropped gels in Source Date. **i,** Side-by-side comparison of the cross-subunit NTD-CTD dimerization interfaces in EcmrR and BmrR dimers. Source data are provided as a Source Data file.

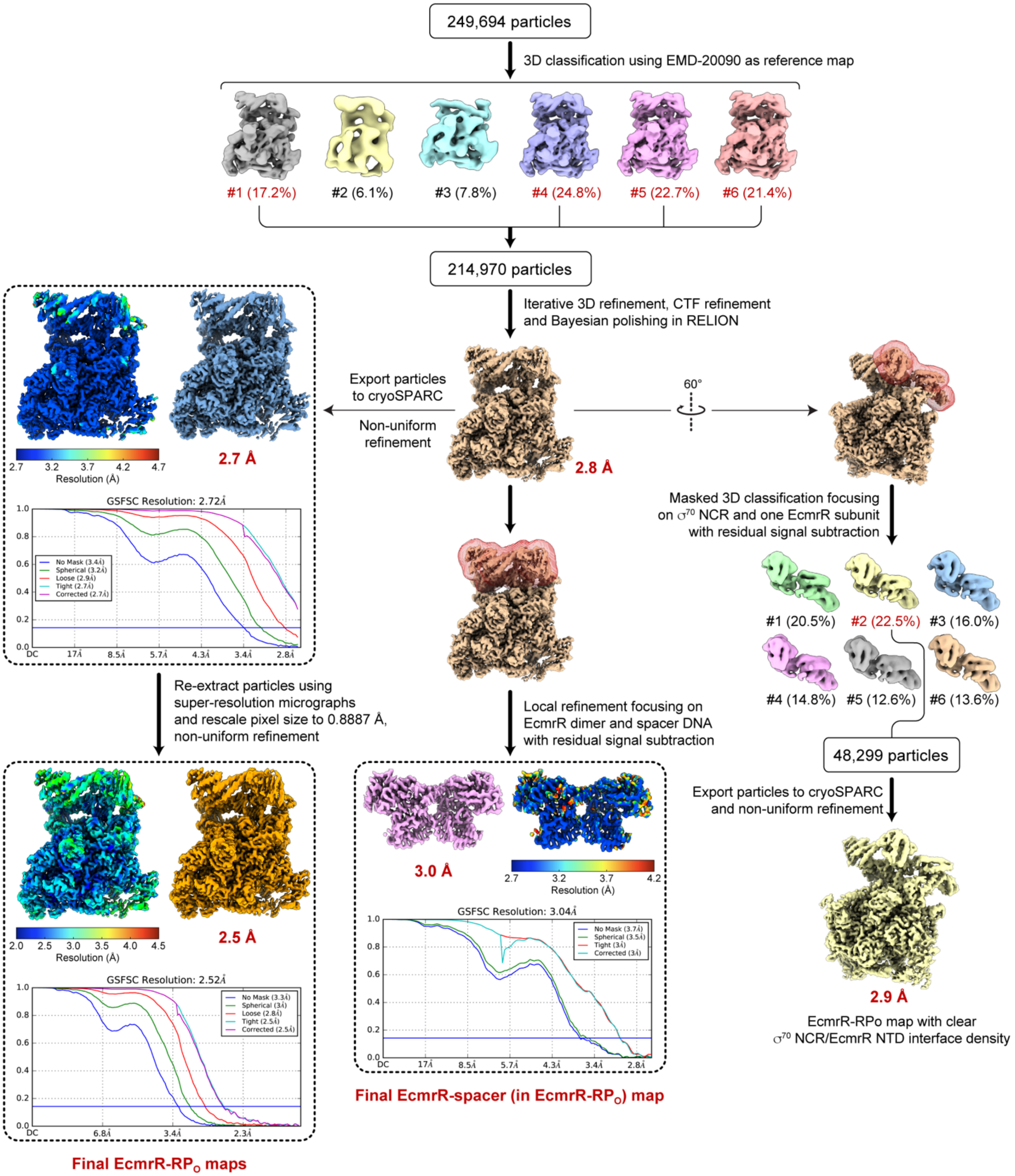

249,694 particles
3D classification using EMD-20090 as reference map
#1 (17.2%)
#2 (6.1%)
#3 (7.8%)
#4 (24.8%)
#5 (22.7%)
#6 (21.4%)
214,970 particles
Iterative 3D refinement, CTF refinement and Bayesian polishing in RELION
Export particles to cryoSPARC
Non-uniform refinement
2.7 Å
Resolution (Å)
GSFSC Resolution: 2.72Å
2.8 Å
60°
Masked 3D classification focusing on σ70 NCR and one EcmrR subunit with residual signal subtraction
#1 (20.5%)
#2 (22.5%)
#3 (16.0%)
#4 (14.8%)
#5 (12.6%)
#6 (13.6%)
Re-extract particles using super-resolution micrographs and rescale pixel size to 0.8887 Å, non-uniform refinement
Local refinement focusing on EcmrR dimer and spacer DNA with residual signal subtraction
48,299 particles
Export particles to cryoSPARC and non-uniform refinement
2.5 Å
GSFSC Resolution: 2.52Å
3.0 Å
GSFSC Resolution: 3.04Å
2.9 Å
EcmrR-RPo map with clear σ70 NCR/EcmrR NTD interface density
Final EcmrR-spacer (in EcmrR-RPO) map
Final EcmrR-RPO maps

Supplementary Fig. 2: Single-particle cryo-EM analysis of EcmrR-RP$_o$.

Flow chart of cryo-EM image processing and map reconstruction for EcmrR-RNAP-promoter complex prepared with a 54-bp heteroduplex DNA scaffold. 3D refinement using all the particles in good 3D classes generated a 2.7 Å map. The FSC curves indicated that map resolution was limited by the micrograph pixel size. Re-extraction the particles from super-resolution micrographs and following 3D refinement improved the map resolution to 2.5 Å. Local 3D refinement focusing on EcmrR dimer and spacer DNA was used to obtain a 3.0 Å map of this subcomplex with significantly improved density of EcmrR. Further masked 3D classification focusing on σ^{70} NCR together with the proximal EcmrR NTD and CTD generated a 2.9 Å map with clear density of σ^{70} NCR and EcmrR NTD interface. All four maps were used as cross-references during model building. The final maps, half-map FSC curves and accompanying local resolution illustrations are enclosed in the dashed black box.

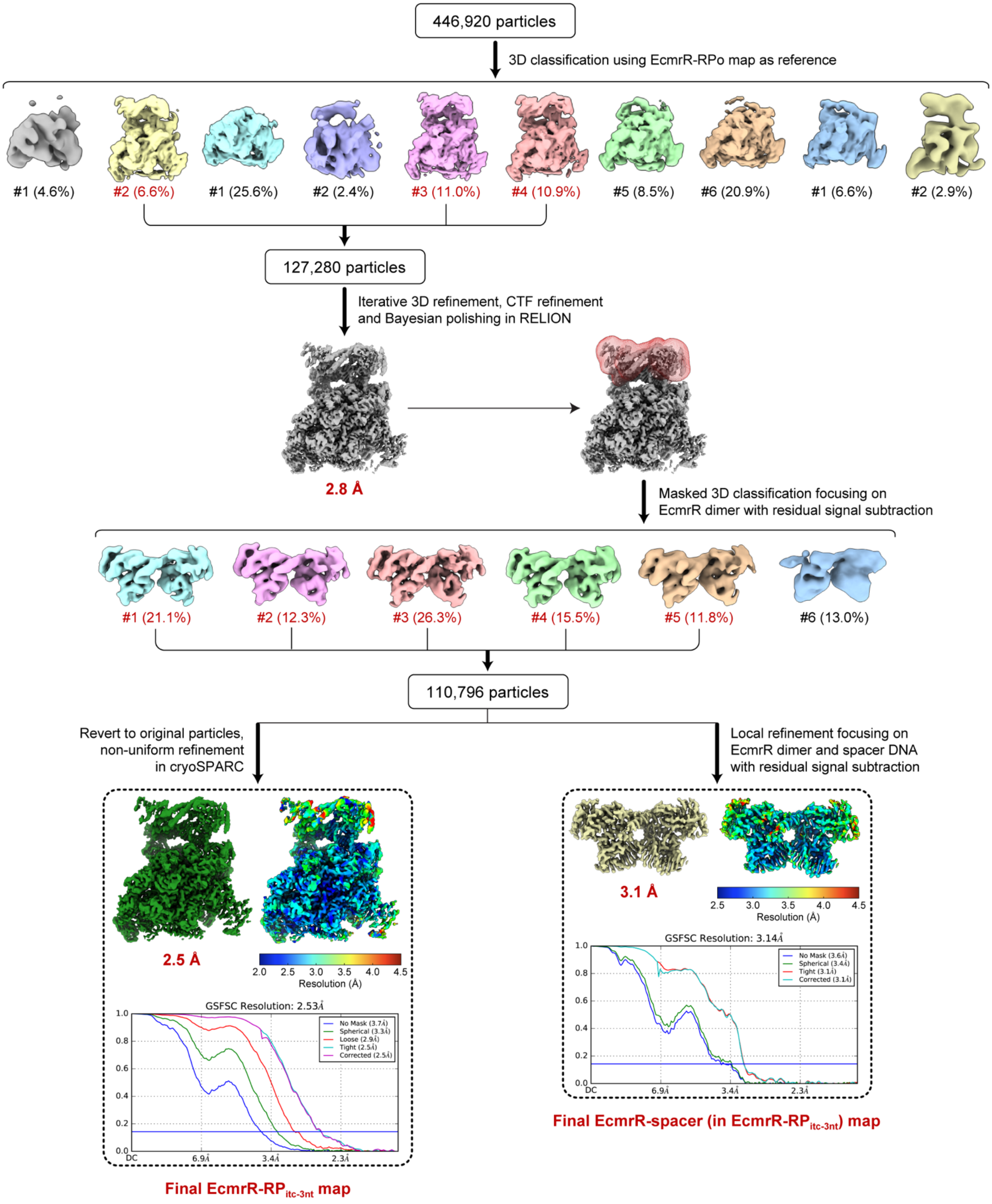
446,920 particles
3D classification using EcmrR-RPo map as reference
#1 (4.6%)
#2 (6.6%)
#1 (25.6%)
#2 (2.4%)
#3 (11.0%)
#4 (10.9%)
#5 (8.5%)
#6 (20.9%)
#1 (6.6%)
#2 (2.9%)
127,280 particles
Iterative 3D refinement, CTF refinement and Bayesian polishing in RELION
2.8 Å
Masked 3D classification focusing on EcmrR dimer with residual signal subtraction
#1 (21.1%)
#2 (12.3%)
#3 (26.3%)
#4 (15.5%)
#5 (11.8%)
#6 (13.0%)
110,796 particles
Revert to original particles, non-uniform refinement in cryoSPARC
Local refinement focusing on EcmrR dimer and spacer DNA with residual signal subtraction
2.5 Å
2.0 2.5 3.0 3.5 4.0 4.5
Resolution (Å)
GSFSC Resolution: 2.53Å
No Mask (3.7Å)
Spherical (3.3Å)
Loose (2.9Å)
Tight (2.5Å)
Corrected (2.5Å)
DC 6.9Å 3.4Å 2.3Å
Final EcmrR-RP$_{itc-3nt}$ map
3.1 Å
2.5 3.0 3.5 4.0 4.5
Resolution (Å)
GSFSC Resolution: 3.14Å
No Mask (3.6Å)
Spherical (3.4Å)
Tight (3.1Å)
Corrected (3.1Å)
DC 6.9Å 3.4Å 2.3Å
Final EcmrR-spacer (in EcmrR-RP$_{itc-3nt}$) map

Supplementary Fig. 3: Single-particle cryo-EM analysis of EcmrR-RP$_{itc-3nt}$.

Flow chart of cryo-EM image processing and map reconstruction for EcmrR-RNAP-promoter complex prepared with a 54-bp heteroduplex DNA scaffold in the presence of ATP and GTP. 3D refinement using all the particles in good 3D classes generated a 2.8 Å map. Masked 3D classification focusing on EcmrR dimer was used to further remove suboptimal particles. Non-uniform refinement of the full complex or local 3D refinement focusing on EcmrR dimer and spacer DNA using the final set of particles generated a 2.5 Å map of the full EcmrR-RP$_{itc-3nt}$ complex or 3.1 Å map of EcmrR dimer with spacer DNA subcomplex, respectively. The two maps were used as cross-references during model building. The final maps, half-map FSC curves and accompanying local resolution illustrations are enclosed in the dashed black box.

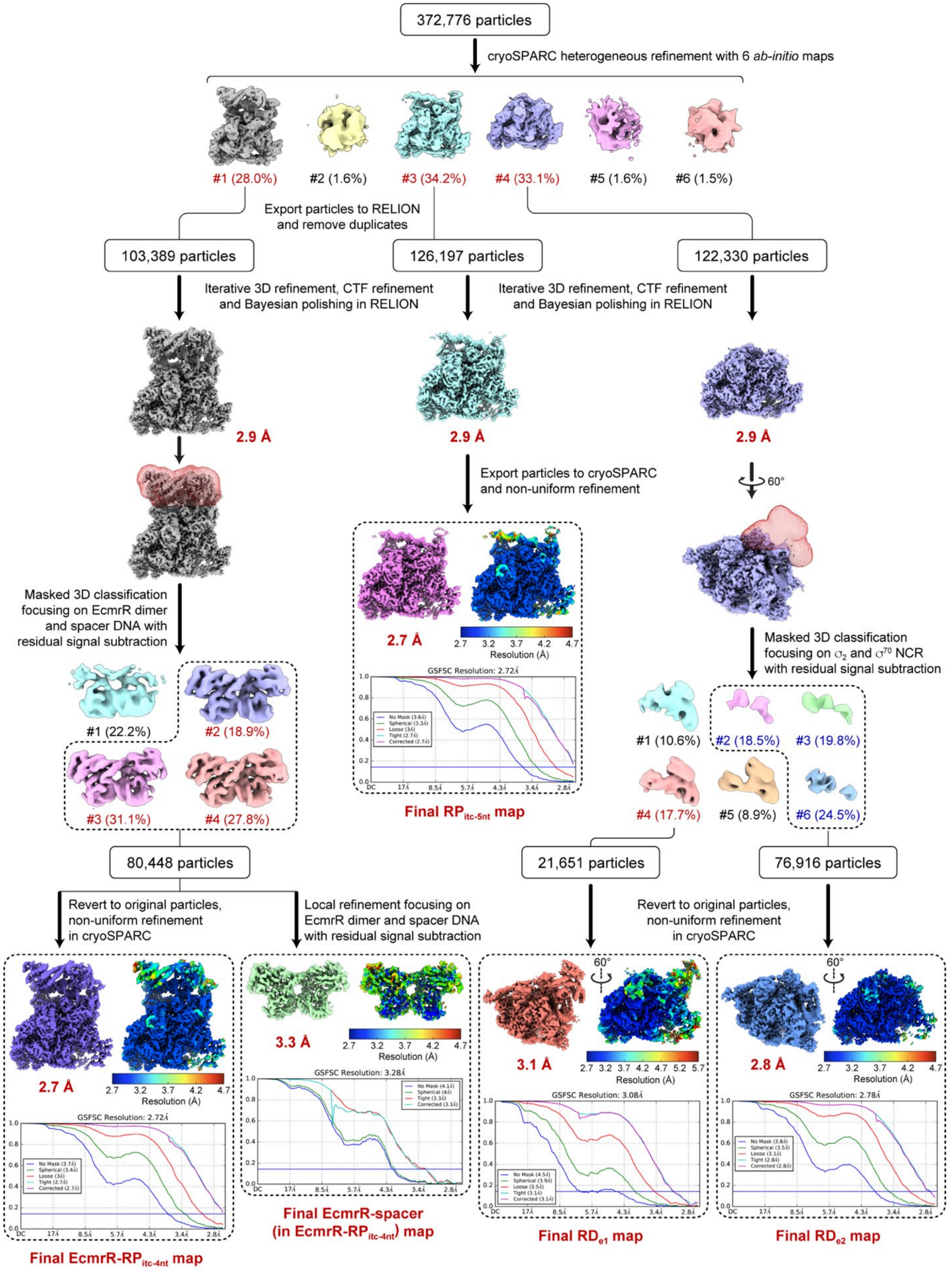
372,776 particles
cryoSPARC heterogeneous refinement with 6 ab-initio maps
#1 (28.0%)
#2 (1.6%)
#3 (34.2%)
#4 (33.1%)
#5 (1.6%)
#6 (1.5%)
Export particles to RELION and remove duplicates
103,389 particles
126,197 particles
122,330 particles
Iterative 3D refinement, CTF refinement and Bayesian polishing in RELION
Iterative 3D refinement, CTF refinement and Bayesian polishing in RELION
2.9 Å
2.9 Å
2.9 Å
Export particles to cryoSPARC and non-uniform refinement
60°
Masked 3D classification focusing on EcmrR dimer and spacer DNA with residual signal subtraction
2.7 Å
Resolution (Å)
GSFSC Resolution: 2.72Å
Final RPitc-5nt map
Masked 3D classification focusing on σ2 and σ70 NCR with residual signal subtraction
#1 (22.2%)
#2 (18.9%)
#3 (31.1%)
#4 (27.8%)
#1 (10.6%)
#2 (18.5%)
#3 (19.8%)
#4 (17.7%)
#5 (8.9%)
#6 (24.5%)
80,448 particles
21,651 particles
76,916 particles
Revert to original particles, non-uniform refinement in cryoSPARC
Local refinement focusing on EcmrR dimer and spacer DNA with residual signal subtraction
Revert to original particles, non-uniform refinement in cryoSPARC
2.7 Å
GSFSC Resolution: 2.72Å
Final EcmrR-RPitc-4nt map
3.3 Å
GSFSC Resolution: 3.28Å
Final EcmrR-spacer (in EcmrR-RPitc-4nt) map
3.1 Å
GSFSC Resolution: 3.08Å
Final RDe1 map
2.8 Å
GSFSC Resolution: 2.78Å
Final RDe2 map

Supplementary Fig. 4: Single-particle cryo-EM analysis of EcmrR-$RP_{itc\text{-}4nt}$, $RP_{itc\text{-}5nt}$, RD_{e1} and RD_{e2}.

Flow chart of cryo-EM image processing and map reconstructions for EcmrR-RNAP-promoter complex reconstituted using a 54-bp fully complementary DNA scaffold in the presence of all four rNTPs. Heterogeneous refinement with six *ab-initio* models generated three major 3D classes with distinct features. Different subsets of particle images were selected from different classification schemes to generate four refined cryo-EM maps: final EcmrR-$RP_{itc\text{-}4nt}$ map at 2.7 Å, final $RP_{itc\text{-}5nt}$ map at 2.7 Å, final RD_{e1} map at 3.1 Å and final RD_{e2} map at 2.8 Å. Local 3D refinement focusing on EcmrR dimer and spacer DNA was used to produce a 3.3 Å local map from the final EcmrR-$RP_{itc\text{-}4nt}$ particle stacks. The local map and the full EcmrR-$RP_{itc\text{-}4nt}$ map were used as cross-references during model building. The final maps, half-map FSC curves and accompanying local resolution illustrations are enclosed in the dashed black box.

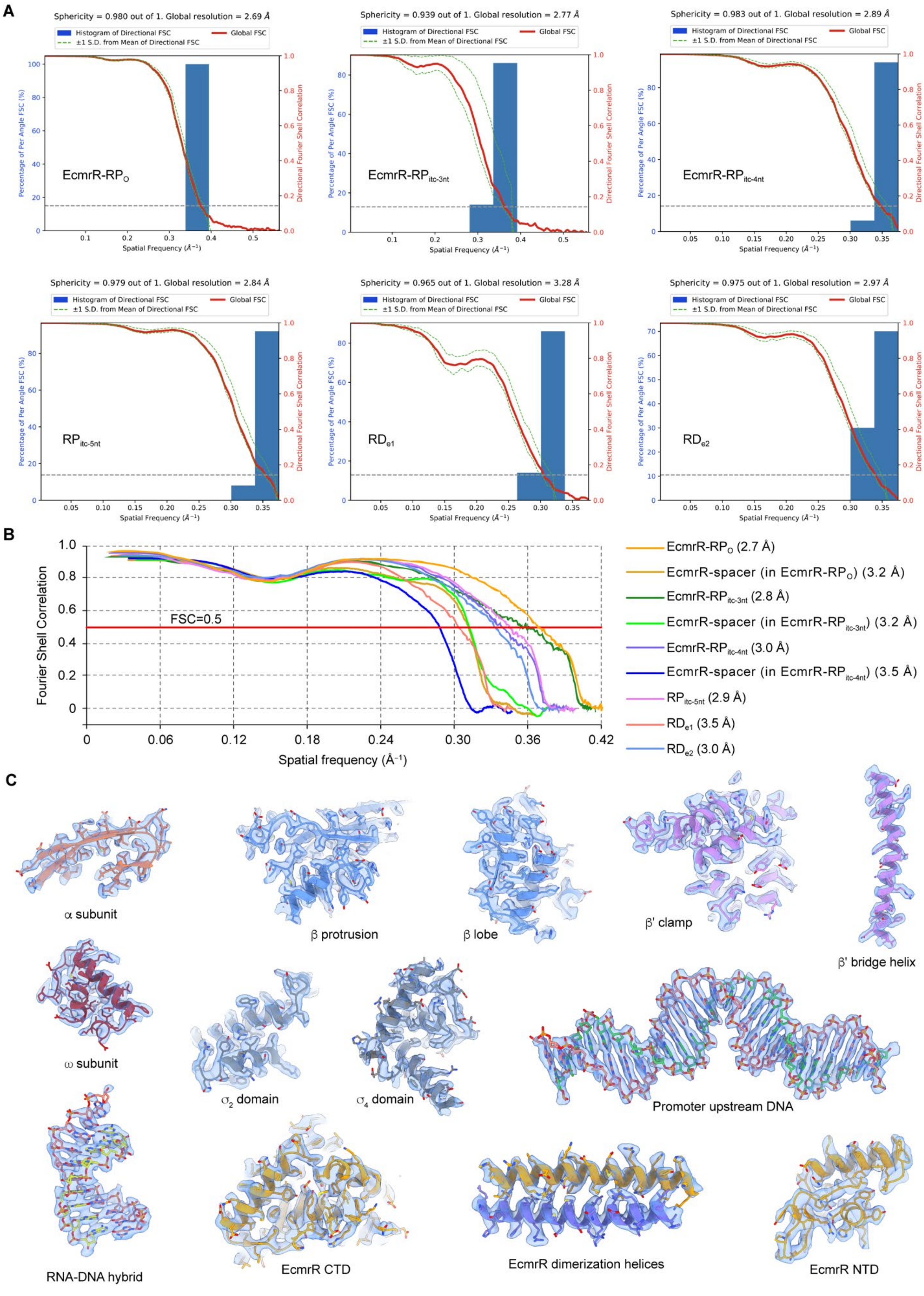
A
Sphericity = 0.980 out of 1. Global resolution = 2.69 Å
Sphericity = 0.939 out of 1. Global resolution = 2.77 Å
Sphericity = 0.983 out of 1. Global resolution = 2.89 Å
Sphericity = 0.979 out of 1. Global resolution = 2.84 Å
Sphericity = 0.965 out of 1. Global resolution = 3.28 Å
Sphericity = 0.975 out of 1. Global resolution = 2.97 Å
Histogram of Directional FSC
Global FSC
±1 S.D. from Mean of Directional FSC
Percentage of Per Angle FSC (%)
Directional Fourier Shell Correlation
Spatial Frequency (Å⁻¹)
EcmrR-RP$_O$
EcmrR-RP$_{itc-3nt}$
EcmrR-RP$_{itc-4nt}$
RP$_{itc-5nt}$
RD$_{e1}$
RD$_{e2}$
B
Fourier Shell Correlation
FSC=0.5
Spatial frequency (Å⁻¹)
EcmrR-RP$_O$ (2.7 Å)
EcmrR-spacer (in EcmrR-RP$_O$) (3.2 Å)
EcmrR-RP$_{itc-3nt}$ (2.8 Å)
EcmrR-spacer (in EcmrR-RP$_{itc-3nt}$) (3.2 Å)
EcmrR-RP$_{itc-4nt}$ (3.0 Å)
EcmrR-spacer (in EcmrR-RP$_{itc-4nt}$) (3.5 Å)
RP$_{itc-5nt}$ (2.9 Å)
RD$_{e1}$ (3.5 Å)
RD$_{e2}$ (3.0 Å)
C
α subunit
β protrusion
β lobe
β' clamp
β' bridge helix
ω subunit
σ$_2$ domain
σ$_4$ domain
Promoter upstream DNA
RNA-DNA hybrid
EcmrR CTD
EcmrR dimerization helices
EcmrR NTD

Supplementary Fig. 5: Validation of cryo-EM structural models.

a Histogram and directional FSC plots for the cryo-EM maps. The sphericity value of each map is indicated. **b** Model-map FSC curves of nine cryo-EM maps and their corresponding atomic models from this study were generated from PHENIX comprehensive validation results. The model-map resolution for each atomic model and its cryo-EM map at FSC = 0.5 cutoff is indicated in the figure and reported in Table 1. **c** Cryo-EM densities superimposed on the atomic model for representative regions of the EcmrR-RNAP-promoter complexes. Cryo-EM densities on RNAP and nucleic acids are contoured at 6σ. Cryo-EM densities on EcmrR are contoured at 12σ.

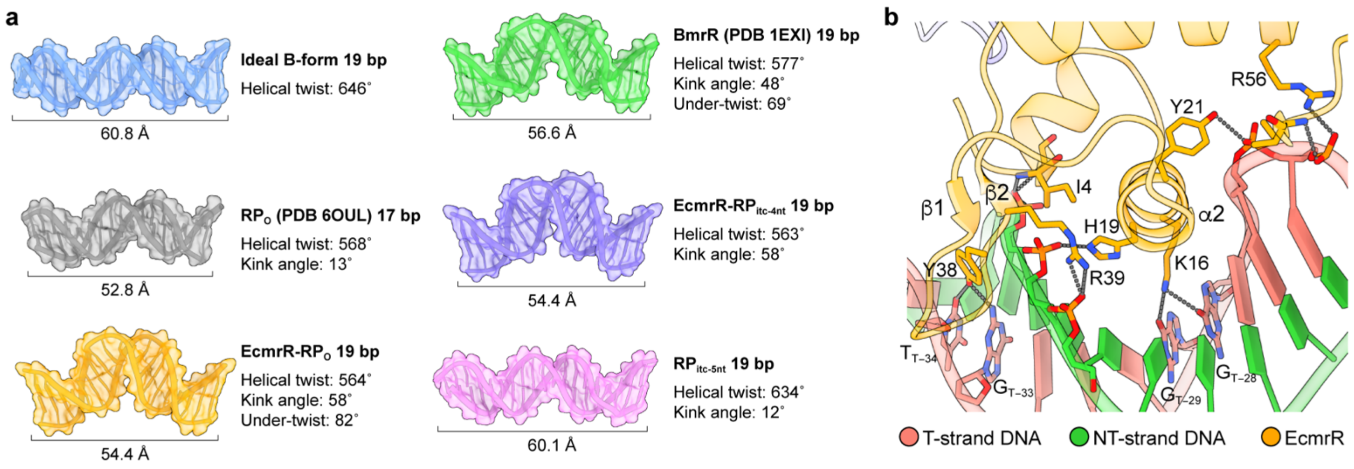

Supplementary Fig. 6: EcmrR binds to and remodels promoter DNA during open complex formation and initial transcription.

a Structural analysis of promoter spacer DNA from various structures. The spacer length, backbone kink angle, total helical twist and under-twist values of the spacer DNA are indicated. For the definitions and measurements of spacer length, backbone kink angle, total helical twist, and under-twist, see METHOD DETAILS. The ideal B-form 19-bp DNA structure was created by W3DNA 2.0 web server. **b** Detailed interactions between EcmrR NTD and promoter spacer DNA. Hydrogen bonds and salt bridges are shown as gray dotted lines.

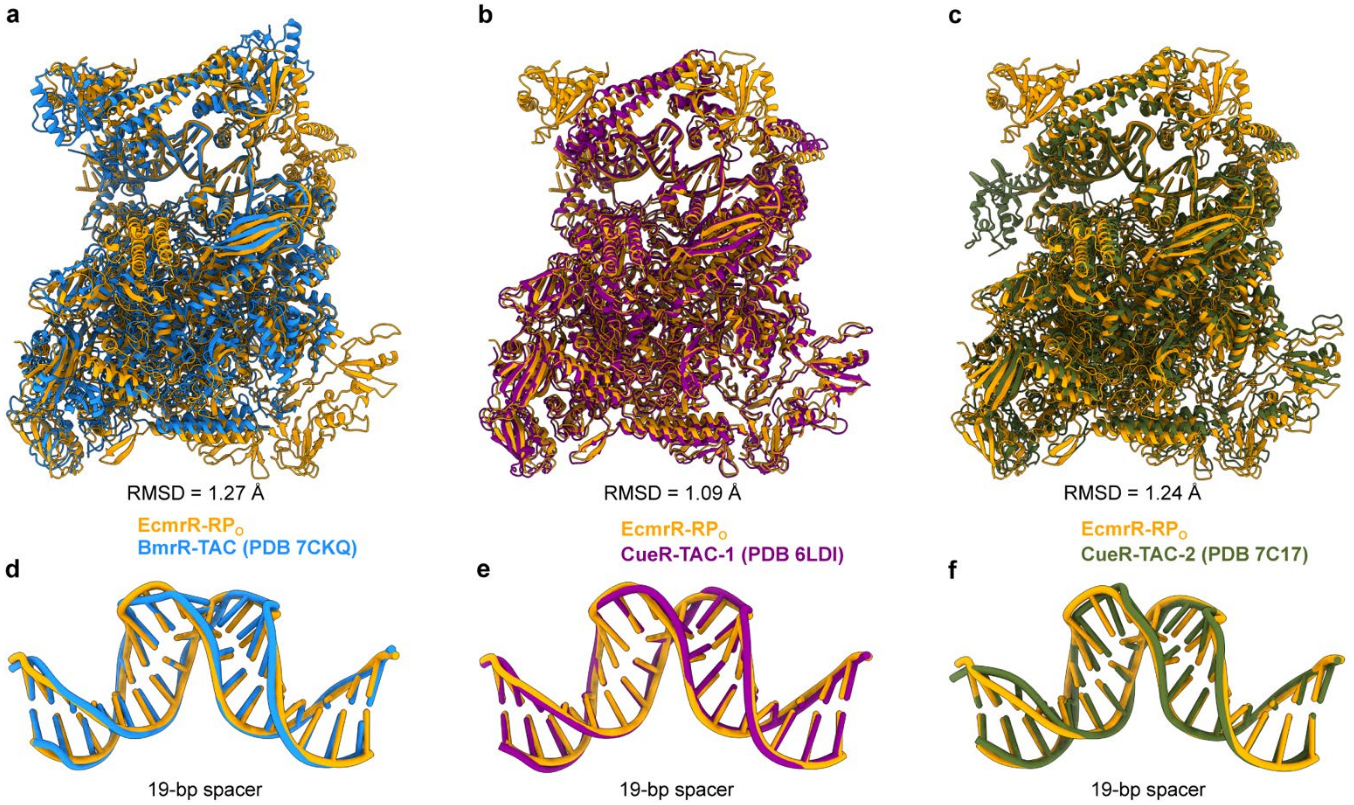

Supplementary Fig. 7: Structural comparison of RNAP in complex with different MerR family transcription regulators.

a Superimposition of EcmrR-RP$_o$ structure with BmrR-TAC structure (PDB 7CKQ). b Superimposition of EcmrR-RP$_o$ structure with CueR-TAC-1 structure (PDB 6LDI). c Superimposition of EcmrR-RP$_o$ structure with CueR-TAC-2 structure (PDB 7C17). **d–f** Close-up views of the 19-bp spacer DNA in the structural superimpositions shown in panels **a–c**. The 19-bp spacers in all four structures exhibit similar level of shortening and kink.

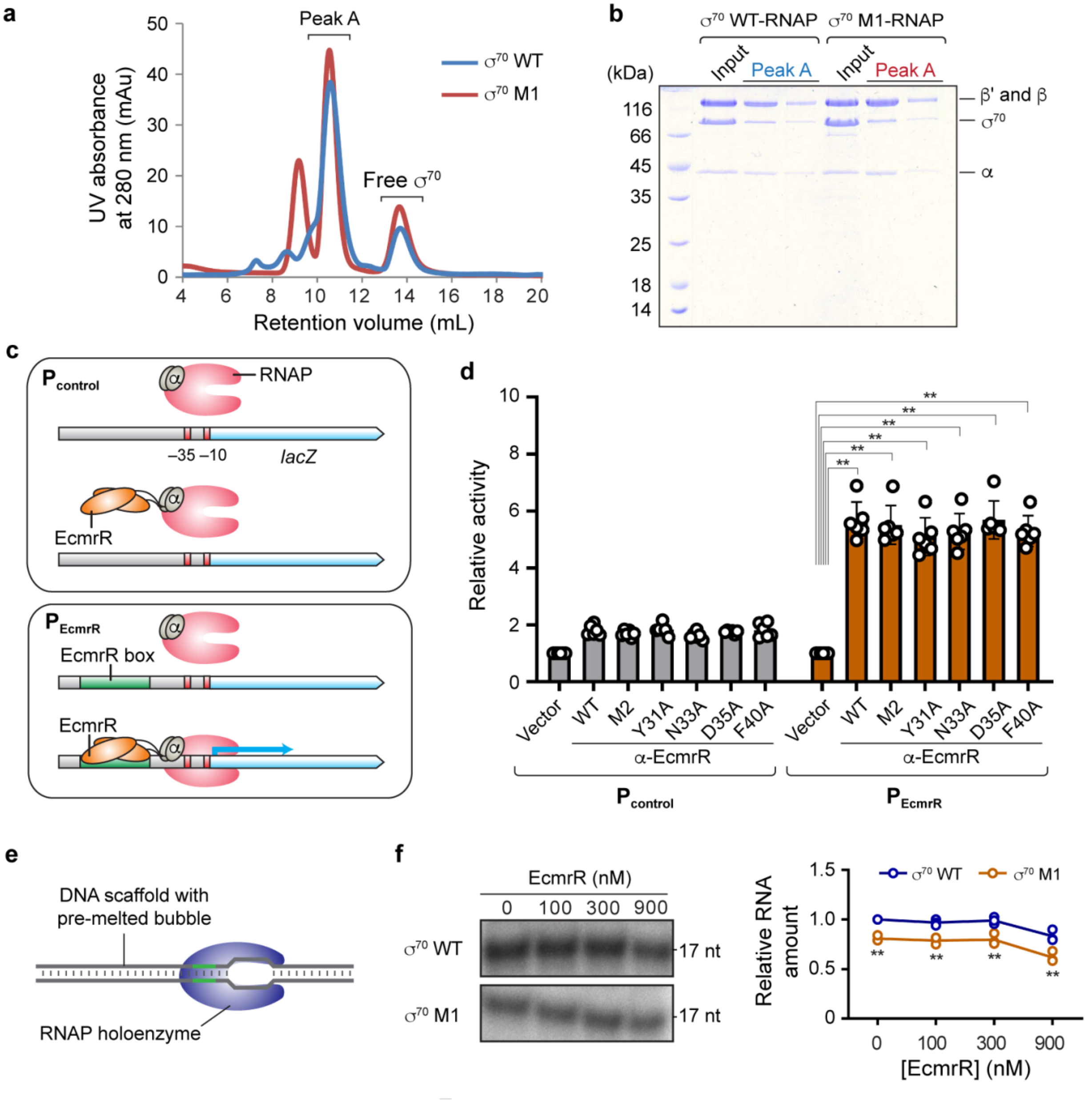

Supplementary Fig. 8: Characterization of mutations at the EcmrR NTD-σ^{70} NCR interface. **a** Size exclusion chromatography (SEC) profiles of RNAP holoenzyme reconstituted using either σ^{70} WT or σ^{70} M1. Peak A corresponds to holoenzyme fractions. **b** The protein compositions in the input fraction and the "Peak A" fractions from each RNAP holoenzyme reconstitution test are analyzed by SDS-PAGE. A representative data from two independent tests is shown. **c** Diagram of the bacterial one hybrid system used in this study to test the DNA-binding abilities of EcmrR

mutants. *lacZ* gene is under the control of a weak promoter and hence has low expression. Binding of EcmrR, which is fused to the C-terminus of RNAP α subunit, to its target site (labeled as EcmrR box) upstream of the weak promoter will help recruit RNA polymerase to the promoter and activate the expression of *lacZ*. **d** Fusing EcmrR to RNAP α subunit greatly increased the expression of *lacZ* in constructs containing EcmrR box upstream of the weak promoter. There is no significant change in *lacZ* expression when mutations at the σ^{70} NCR-interacting interface (Y31A, N33A, D35A, F40A, or the combination of all four mutations, designated EcmrR M2) are introduced in EcmrR, suggesting these mutations do not affect the binding of EcmrR to its target sequence. Data were obtained from three colonies performed in duplicate and are shown as mean ± SEM. **e** Diagram of the *in vitro* transcription assay performed on DNA scaffold containing a 19-bp spacer promoter and pre-melted bubble. EcmrR target sequence is colored in green. **f** *In vitro* transcription on the DNA scaffold shown in panel **e** using *E.coli* RNAP assembled with σ^{70} WT or σ^{70} M1. The concentrations (in nM) of EcmrR are indicated. The experiment was repeated three times and similar results were obtained. RNA products were quantified from these three independent experiments and are shown as mean ± SD. Statistical analyses were performed using the unpaired Student's *t*-test (two-tailed). $^{**}P < 0.01$. Source data are provided as a Source Data file.

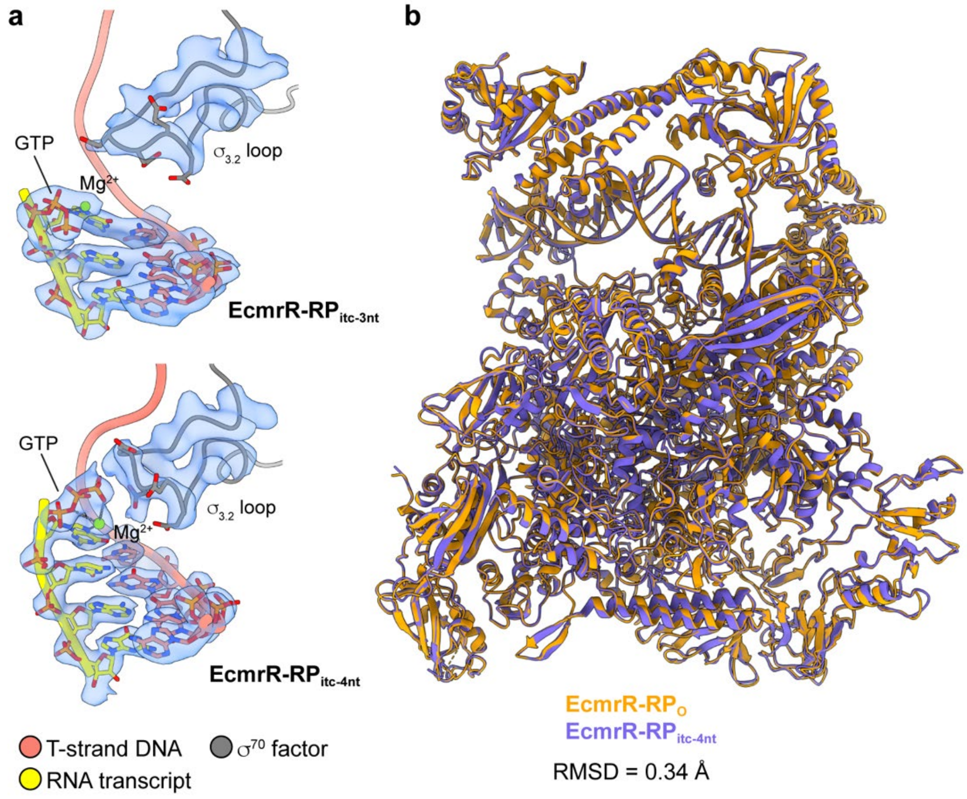

Supplementary Fig. 9: Structural comparison of EcmrR-RNAP-promoter initial transcribing complexes.

a Comparison of $\sigma_{3.2}$ loop conformations in EcmrR-$RP_{itc-3nt}$ and EcmrR-$RP_{itc-4nt}$. The $\sigma_{3.2}$ loop and RNA-DNA duplex from the two structures are superimposed with their respective cryo-EM densities contoured at 5σ. The Mg^{2+} ions coordinated by 5' GTP of the nascent RNA transcripts are shown as green sphere. **b** Superimposition of EcmrR-RP_o structure with EcmrR-$RP_{itc-4nt}$ structure.

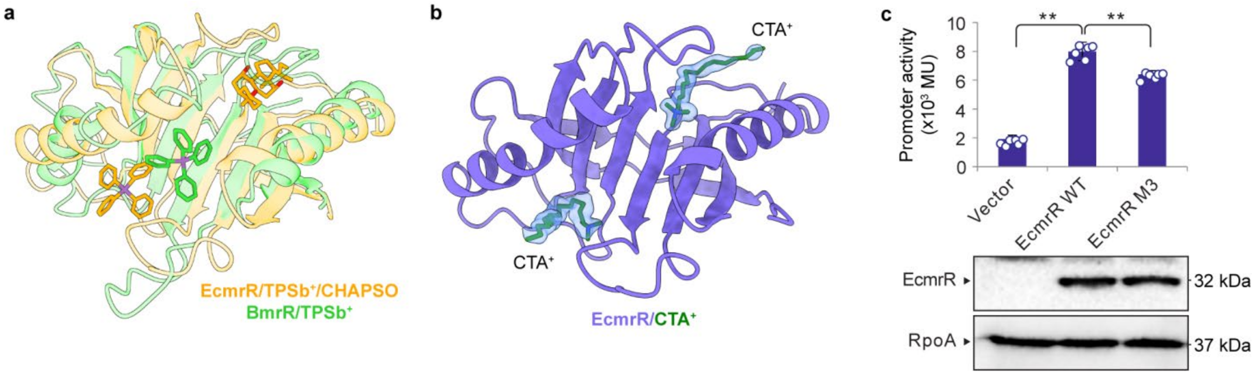

Supplementary Fig. 10: Two ligand-binding sites in EcmrR.

a Comparison of the ligand-binding sites in EcmrR and BmrR. The CTDs of EcmrR and BmrR are superimposed. The drug-like compounds in the two structures are shown as sticks. **b** Each of the two ligand-binding sites in EcmrR CTD crystal structure is bound by a CTA^+ ion. The ligands are superimposed with the F_o–F_c polder omit map[71] contoured at 5σ. **c** Activation of *in vivo* transcription from an EcmrR-dependent promoter by EcmrR WT and EcmrR bearing mutations in the ligand-binding site II (Y174A, designated EcmrR M3). The promoter is fused to a β-galactosidase reporter gene (*lacZ*). Promoter activity was measured by β-galactosidase activity in miller units (MU). Expression levels of EcmrR and the loading control RpoA are shown. The results indicate that the decrease in transcription activation by EcmrR M3 is not due to large decrease in protein expression. Data were obtained from three colonies performed in duplicate and are shown as mean ± SEM. Statistical analyses were performed using the unpaired Student's *t*-test (two-tailed). $^{**}P < 0.01$. Source data are provided as a Source Data file.

Supplementary Table 1: Data collection and refinement statistics.

	EcmrR-CTD native (PDB 6WL5)	EcmrR-CTD Br-derivative	EcmrR-CTD I-derivative
**Data collection**			
Space group	*C*2	*C*2	*C*2
Cell dimensions			
a, *b*, *c* (Å)	212.393, 42.419, 115.798	212.196, 42.382, 115.815	212.119, 42.413, 116.674
α, β, γ (°)	90, 117.27, 90	90, 117.21, 90	90, 117.086, 90
Resolution (Å)	103.21–1.40 (1.45–1.40) *	103.22–1.56 (1.62–1.56)	104.11–1.57 (1.63–1.57)
R_{sym} or R_{merge}	0.049 (0.601)	0.095 (1.587)	0.094 (0.875)
$I / \sigma I$	12.4 (1.7)	12.6 (1.0)	11.4 (1.7)
Completeness (%)	98.4 (99.2)	99.0 (94.9)	97.7 (94.9)
Redundancy	3.4 (3.4)	6.7 (6.2)	6.0 (5.4)
**Refinement**			
Resolution (Å)	102.93–1.40 (1.45–1.40)		
No. reflections	178280 (17844)		
R_{work} / R_{free}	0.1434/0.1734		
No. atoms			
Protein	10845		
Ligand/ion	740		
Water	969		
B-factors			
Protein	20.2		
Ligand/ion	38.4		
Water	37.0		
R.m.s. deviations			
Bond lengths (Å)	0.014		
Bond angles (°)	1.37		

*Values in parentheses are for highest-resolution shell.

†One native crystal, one Br-derivative crystal and one I-derivative crystal were used for structural determination through multiple isomorphous replacement with anomalous scattering (MIRAS) method.

Supplementary Table 2: Primers used in this study.

Name	Sequence (5' to 3')	Source	Application
EcmrR-BamHI-F	AAAAAAGGATCCCAAATCGGTCTGTTCAGCAAAATC	IDT	EcmrR cloning
EcmrR-C-BamHI-F	AAAAAAGGATCCATGCTGTACCAGGCGGC	IDT	
EcmrR-HindIII-R	AAAAAAAAGCTTTTACACCTTCTCCACCGGGAATTG	IDT	
EcmrR-M1-F	CAAATCGCGAGCCTGCGTgcGCTGGGCTTCACCATCACCGAG	TIANYI, China	EcmrR mutation
EcmrR-M1-R	ACGCAGGCTCGCGATTTGagcAAATTTCATCgcCTGATCGCTGGTATAGAAACGGTA	TIANYI, China	
EcmrR-M2-F	CCGGCTAACGGTTACCGTGCTTATACCAGCGATCAGCTGATGAAATTTCACC	TIANYI, China	
EcmrR-M2-R	ACGGTAACCGTTAGCCGGGGCAATAGCCGCCGGAACCAGCAGACCG	TIANYI, China	
EcmrR-Y31A-F	CGCCGGAACCAGCAGACCGATTTTATTGTAGTAATG	TIANYI, China	
EcmrR-Y31A-R	GTCTGCTGGTTCCGGCGGCGATTAACCCGGACAACGGTTACCGTTTCTATACCAGCGAT	TIANYI, China	
EcmrR-N33A-F	GTCTGCTGGTTCCGGCGTATATTGCCCCGGACAACGGTTACCGTTTCTATACCAGCGAT	TIANYI, China	
EcmrR-N33A-R	GTCTGCTGGTTCCGGCGTATATTAACCCGGCCAACGGTTACCGTTTCTATACCAGCGAT	TIANYI, China	
EcmrR-D35A-F	TATACCAGCGATCAGCTGATGAAATTTCACCAAATC	TIANYI, China	
EcmrR-D35A-R	CAGCTGATCGCTGGTATAGGCACGGTAACCGTTGTCCGGGTTA	TIANYI, China	
EcmrR-F40A-F	CGCCGGAACCAGCAGACCGATTTTATTGTAGTAATG	TIANYI, China	
EcmrR-F40A-R	GTCTGCTGGTTCCGGCGGCGATTAACCCGGACAACGGTTACCGTTTCTATACCAGCGAT	TIANYI, China	
EcrpoD-NheI-F	GATATACATATGGCTAGCATGGAGCAAAACCCGCAGTCACAG	TIANYI, China	RpoD mutation
EcrpoD-HindIII-R	GGCCGCAAGCTTATCGTCCAGGAAGCTACGCAGCA	TIANYI, China	
EcrpoD-M1-F1	GAAGAAGCGGCGCTGGCAGACCTGATCACCGGCTTTGTTGACCCG	TIANYI, China	
EcrpoD-M1-R1	GCCAGCGCCGCTTCTTCTGCTTCAACACGATCGTACTGTTC	TIANYI, China	
EcrpoD-M1-F2	AGTTCCGCCTGGTGCCGGCACAGTTTGACTACCTGGTCAACAG	TIANYI, China	
EcrpoD-M1-R2	CGGCACCAGGCGGAACTGTTTGAATAC	TIANYI, China	
PEcmrR-lacZ-F	GGTCCATCTATTAATTCAATTATAACTTCTCTCTAACGCTGTGTATCG	TIANYI, China	Bacterial one-hybrid system
PEcmrR-lacZ-R	ATTGAATTAATAGATGGACCCTCCCCTTAGGGGAGGGTAATCGGGTGAAAACATCGGC	TIANYI, China	
pRAT103-F	GGTACCTTAAGCCAGCCCCGACAC	TIANYI, China	
pRAT103-R	CTGCAGACTAGTGGCCGCCTCTGGTTTCTC	TIANYI, China	
pRAT-EcmrR-F	GCGGCCACTAGTCTGCAGATCGGTCTGTTCAGCAAAATCTGC	TIANYI, China	

pRAT-EcmrR-R	GGGCTGGCTTAAGGTACCTTACACCTTCTCCACCGGGAATTGA	TIANYI, China	
NT strand DNA-1	GCCTTGACCCTCCCCTAAGGGGAGGGTTTAGATTGTGTGCAGTCTGACGCGGCG	IDT	TAC Complex assembly
T strand DNA-1	CGCCGCGTCAGACTCGTAGGAATCTAAACCCTCCCCTTAGGGGAGGGTCAAGGC	IDT	
T strand DNA-2	CGCCGCGTCAGACTGCACACAATCTAAACCCTCCCCTTAGGGGAGGGTCAAGGC	IDT	
PEcmrR-F2	CCGGATGCAAATCGAGCCGATTTTTTAATCTTTACGGACTTTTACCCGCCTGGTTTATTAATTTCTTGACC	TIANYI, China	*In vitro* transcription template
PEcmrR-R2	CCGCGCACTCCTTTAAGACAGTTTTGACTGGCTGCACACAATCTAAACCCTCCCCTTAGGGGAGGGTCAAGAAATTAATAAACCAGG	TIANYI, China	
PEcmrR-PreO-F	GCCTTGACCCTCCCCTAAGGGGAGGGTTTAGATTGTATGCTCAGTGTATCCCGGGCG	TIANYI, China	Pre-melted promoter for *in vitro* transcription
PEcmrR-PreO-R	CGCCCGGGATACACACTCGTAGGAATCTAAACCCTCCCCTTAGGGGAGGGTCAAGGC	TIANYI, China	

Supplementary Video 1: A composite morph of structural transitions during EcmrR-dependent transcription. EcmrR, RNAP subunits, nucleic acids are colored in the same way as they are in Fig. 1 and Fig. 3. Names of the structures are indicated.
