# Supplementary material for: Structural visualization of transcription activated by a multidrug-sensing MerR family regulator
Source: Nat Commun. 2021 May 11;12:2702. doi: 10.1038/s41467-021-22990-8 (PMC8113463; doi:10.1038/s41467-021-22990-8)
Supplement: Supplementary file 3 — Description of Additional Supplementary Files [file 41467_2021_22990_MOESM3_ESM.docx]

Description of additional supplementary information

Title: Supplementary Video 1

Description: A composite morph of structural transitions during EcmrR-dependent transcription. EcmrR, RNAP subunits, nucleic acids are colored in the same way as they are in Fig. 1 and Fig. 3. Names of the structures are indicated.
