# Supplementary material for: Structural visualization of transcription activated by a multidrug-sensing MerR family regulator
Source: Nat Commun. 2021 May 11;12:2702. doi: 10.1038/s41467-021-22990-8 (PMC8113463; doi:10.1038/s41467-021-22990-8)
Supplement: Supplementary file 4 — Reporting Summary [file 41467_2021_22990_MOESM4_ESM.pdf]

nature research

Corresponding author(s): Yang Yang, Chang Liu, Yangbo Hu, Bin Liu

Last updated by author(s): Mar 27, 2021

Reporting Summary

Nature Research wishes to improve the reproducibility of the work that we publish. This form provides structure for consistency and transparency in reporting. For further information on Nature Research policies, see our Editorial Policies and the Editorial Policy Checklist.

Statistics

For all statistical analyses, confirm that the following items are present in the figure legend, table legend, main text, or Methods section.

n/a	Confirmed	
☐	☒	The exact sample size (*n*) for each experimental group/condition, given as a discrete number and unit of measurement
☐	☒	A statement on whether measurements were taken from distinct samples or whether the same sample was measured repeatedly
☐	☒	The statistical test(s) used AND whether they are one- or two-sided<br>*Only common tests should be described solely by name; describe more complex techniques in the Methods section.*
☒	☐	A description of all covariates tested
☒	☐	A description of any assumptions or corrections, such as tests of normality and adjustment for multiple comparisons
☐	☒	A full description of the statistical parameters including central tendency (e.g. means) or other basic estimates (e.g. regression coefficient) AND variation (e.g. standard deviation) or associated estimates of uncertainty (e.g. confidence intervals)
☐	☒	For null hypothesis testing, the test statistic (e.g. *F*, *t*, *r*) with confidence intervals, effect sizes, degrees of freedom and *P* value noted<br>*Give P values as exact values whenever suitable.*
☒	☐	For Bayesian analysis, information on the choice of priors and Markov chain Monte Carlo settings
☒	☐	For hierarchical and complex designs, identification of the appropriate level for tests and full reporting of outcomes
☒	☐	Estimates of effect sizes (e.g. Cohen's *d*, Pearson's *r*), indicating how they were calculated

Our web collection on statistics for biologists contains articles on many of the points above.

Software and code

Policy information about availability of computer code

Data collection	SerialEM version 3.6; EPU version 2.5
Data analysis	RELION-3.1; MotionCor2; CTFFIND-4.1.13; Gautomatch 0.56; cryoSPARC v2.14; UCSF pyem v0.5; ISOLDE v1.0b4; Phenix-1.16; Coot-0.8.9; UCSF ChimeraX 0.93; XDS version March 2018; SHELXD-2013; AutoSol, AutoBuild and MolProbity included in Phenix-1.16; 3DFSC web server; PROMALS3D web server; Espript 3.0 web server; W3DNA 2.0 web server; ImageJ 1.44p

For manuscripts utilizing custom algorithms or software that are central to the research but not yet described in published literature, software must be made available to editors and reviewers. We strongly encourage code deposition in a community repository (e.g. GitHub). See the Nature Research guidelines for submitting code & software for further information.

Data

Policy information about availability of data

All manuscripts must include a data availability statement. This statement should provide the following information, where applicable:

- Accession codes, unique identifiers, or web links for publicly available datasets
- A list of figures that have associated raw data
- A description of any restrictions on data availability

Atomic coordinates of ten structures have been deposited in PDB with accession numbers 6WL5 (https://www.rcsb.org/structure/6WL5) (EcmrR CTD), 6XL5 (https://www.rcsb.org/structure/6XL5) (EcmrR-RPo), 6XL6 (https://www.rcsb.org/structure/6XL6) (EcmrR-spacer DNA complex from EcmrR-RPo), 6XL9 (https://www.rcsb.org/structure/6XL9) (EcmrR-RPitc-3nt), 6XLA (EcmrR-spacer DNA complex from EcmrR-RPitc-3nt), 6XLJ (https://www.rcsb.org/structure/6XLJ) (EcmrR-RPitc-4nt), 6XLK (https://www.rcsb.org/structure/6XLK) (EcmrR-spacer DNA complex from EcmrR-RPitc-4nt), 6XLL (https://www.rcsb.org/structure/6XLL) (RPitc-5nt), 6XLM (https://www.rcsb.org/structure/6XLM) (RDe1) and 6XLN (https://www.rcsb.org/structure/6XLN) (RDe2). Ten cryo-EM density maps of different EcmrR and RNAP complexes have been deposited in the Electron Microscopy Data Bank with accession number EMD-22234 (https://www.ebi.ac.uk/pdbe/entry/

emdb/EMD-22234) (EcmrR-RPo), EMD-23291 (https://www.ebi.ac.uk/pdbe/entry/emdb/EMD-23291) (EcmrR-RPo with clear σ70 NCR-EcmrR NTD interface density), EMD-22235 (https://www.ebi.ac.uk/pdbe/entry/emdb/EMD-22235) (EcmrR-spacer DNA complex from EcmrR-RPo), EMD-22236 (https://www.ebi.ac.uk/pdbe/entry/emdb/EMD-22236) (EcmrR-RPitc-3nt), EMD-22237 (https://www.ebi.ac.uk/pdbe/entry/emdb/EMD-22237) (EcmrR-spacer DNA complex from EcmrR-RPitc-3nt), EMD-22245 (https://www.ebi.ac.uk/pdbe/entry/emdb/EMD-22245) (EcmrR-RPitc-4nt), EMD-22246 (https://www.ebi.ac.uk/pdbe/entry/emdb/EMD-22246) (EcmrR-spacer DNA complex from EcmrR-RPitc-4nt), EMD-22247 (https://www.ebi.ac.uk/pdbe/entry/emdb/EMD-22247) (RPitc-5nt), EMD-22248 (https://www.ebi.ac.uk/pdbe/entry/emdb/EMD-22248) (RDe1), EMD-22249 (https://www.ebi.ac.uk/pdbe/entry/emdb/EMD-22249) (RDe2), respectively. Source data are provided with this paper.

Field-specific reporting

Please select the one below that is the best fit for your research. If you are not sure, read the appropriate sections before making your selection.

☒ Life sciences ☐ Behavioural & social sciences ☐ Ecological, evolutionary & environmental sciences

For a reference copy of the document with all sections, see nature.com/documents/nr-reporting-summary-flat.pdf

Life sciences study design

All studies must disclose on these points even when the disclosure is negative.

Sample size	No statistical methods were used to predetermine sample size. Sufficient cryo-EM raw movies were collected until cryo-EM maps with adequate resolutions for model building can be achieved. For functional assays, the number of times that experiments were repeated is addressed under "Replication" below.
Data exclusions	No data were excluded from analyses.
Replication	All biochemical and in vivo transcription assays were repeated three times. Each replicate was an independent experiment and did not represent re-assay of the same material. All attempts at replication were successful.
Randomization	Randomization was not relevant to our study because our study did not involve the allocation of samples/organisms/participants into experimental groups.
Blinding	Investigators were not blinded to group allocation because group allocation was not involved in our study. Investigators were not blinded during data collection because the data being collected were quantitative in nature (gels or numbers of colonies on a plate) and were not prone to subjective interpretation.

Reporting for specific materials, systems and methods

We require information from authors about some types of materials, experimental systems and methods used in many studies. Here, indicate whether each material, system or method listed is relevant to your study. If you are not sure if a list item applies to your research, read the appropriate section before selecting a response.

Materials & experimental systems

n/a	Involved in the study	
☐	☒	Antibodies
☒	☐	Eukaryotic cell lines
☒	☐	Palaeontology and archaeology
☒	☐	Animals and other organisms
☒	☐	Human research participants
☒	☐	Clinical data
☒	☐	Dual use research of concern

Methods

n/a	Involved in the study	
☒	☐	ChIP-seq
☒	☐	Flow cytometry
☒	☐	MRI-based neuroimaging

Antibodies

Antibodies used	Rabbit anti-His antibody (ABBkine A02051, 1:2000 dilution in Blocking Buffer (0.5% Tween-20 in 1x PBS); Rabbit anti-RpoA serum was made in house using purified RpoA protein, 1:1000 dilution in Blocking Buffer; HRP-labeled Goat Anti-Rabbit IgG (Beyotime A0208, 1:10000 dilution in Blocking Buffer).
Validation	Rabbit anti-His antibody was validated by manufacturer by western blot against His-tag fusion protein in cell lysate (https://www.abbkine.com/product/anti-his-tag-rabbit-polyclonal-antibody-a02051/); Rabbit anti-RpoA serum was validated by western blot against E. coli cell lysate showing only one major band of the expected molecular weight; HRP-labeled Goat Anti-Rabbit IgG was validated by western blot against rabbit primary antibodies that target specific proteins from E. coli cell lysates. The specificity of this antibody are also validated by other studies listed on the manufacturer's website (https://www.beyotime.com/product/A0208.htm).
